# Comparative phytochemical, antioxidant, and hemostatic studies of fractions from raw and roasted sea buckthorn seeds in vitro

**DOI:** 10.1038/s41598-024-72012-y

**Published:** 2024-09-11

**Authors:** Natalia Sławińska, Jerzy Żuchowski, Anna Stochmal, Beata Olas

**Affiliations:** 1 Department of General Biochemistry, Faculty of Biology and Environmental Protection, University of Lodz, 90-236 Lodz, Poland; 2 Department of Biochemistry and Crop Quality, Institute of Soil Science and Plant Cultivation – State Research Institute, Czartoryskich 8, 24-100 Puławy, Poland

**Keywords:** Oxidative stress, Plasma, Sea buckthorn, Seeds, Thermal processing, Biochemistry, Plant sciences

## Abstract

Various seeds, including sea buckthorn (*Hippophae rhamnoides* L.) seeds, are sources of different bioactive compounds. They can show anti-inflammatory, hypoglycemic, anti-hyperlipidemic, antibacterial, antioxidant, or other biological properties in in vitro and in vivo models. Our preliminary in vitro results have demonstrated that the extracts from raw (no thermal processing) and roasted (thermally processed) sea buckthorn seeds have antioxidant potential and anticoagulant activity. However, it was unclear which compounds were responsible for these properties. Therefore, in continuation of our previous study, the extracts were fractionated by C18 chromatography. Phytochemical analysis of three fractions (a, b, and c) from raw sea buckthorn seeds and four fractions (d, e, f, and g) from roasted sea buckthorn seeds were performed. Several in vitro assays were also conducted to determine the antioxidant and procoagulant/anticoagulant potential of the fractions and two of their major constituents—isorhamnetin 3-*O*-β-glucoside7-*O*-α-rhamnoside and serotonin. LC–MS analyses showed that serotonin is the dominant constituent of fractions c and f, which was tentatively identified on the basis of its HRMS and UV spectra. Moreover, fractions c and f, as well as b and e, contained different B-type proanthocyanidins. Fractions b and e consisted mainly of numerous glycosides of kaempferol, quercetin, and isorhamnetin. The results of oxidative stress assays (measurements of protein carbonylation, lipid peroxidation, and thiol groups oxidation) showed that out of all the tested fractions, fraction g (isolated from roasted seeds and containing mainly dihexoses, and serotonin) demonstrated the strongest antioxidant properties.

## Introduction

Plant seeds, including sea buckthorn (*Hippophae rhamnoides* L*.*, also classified as *Elaegnus rhamnoides* (L.) A. Nelson) seeds, are sources of diverse bioactive compounds^1–3^. Several studies have suggested that increased seed consumption is associated with decreased prevalence of various diseases, such as cardiovascular diseases (CVDs), which are tied to oxidative stress and changes in hemostasis^4–7^. Our previous study demonstrated that sea buckthorn seed extract contains diverse secondary metabolites, mainly glycosides of isorhamnetin, kaempferol, and quercetin^8^. In addition, researchers have shown that sea buckthorn seed extracts have anti-inflammatory, hypoglycemic, anti-hyperlipidemic, antibacterial, and antioxidant activities (among others), which were studied in both in vitro and in vivo models^9–12^. These results show that using sea buckthorn seeds might bring a range of beneficial health effects.

Seeds, as a component of food, are often exposed to high temperatures. Thermal processing can affect the content of phenolic compounds and other phytochemicals in different ways. The content of phenolics can increase or decrease. High temperatures can also change their biological activity; for example, antioxidant potential can improve or diminish during thermal processing^13^. Our preliminary in vitro results have shown that the extracts from raw (no thermal processing) and roasted (thermally processed) sea buckthorn seeds have antioxidant potential and anticoagulant activity. In addition, we have demonstrated that the composition of the extract from raw seeds does not differ significantly from the extract from roasted seeds—both extracts contained various phenolic compounds, including flavonoids (glycosides of quercetin, kaempferol, and isorhamnetin), B-type pro anthocyanidins, and catechins^8^. However, it was unclear which compounds are responsible for their biological activity. Therefore, in continuation of our previous study, we analyzed the phytochemical content of three fractions (a, b, and c) from raw sea buckthorn seed extract and four fractions (d, e, f, and g) from roasted sea buckthorn seed extract. Each fraction contained a separate group of compounds. We also estimated their antioxidant and procoagulant/anticoagulant potential in vitro. Moreover, we determined the antioxidant potential of two chemical compounds (isorhamnetin 3-*O*-β-glucoside7-*O*-α-rhamnoside and serotonin (5-hydroxytryptamine, 5-HT)) present in the fractions; ascorbic acid (vitamin C) was used as a reference antioxidant. We measured three parameters of oxidative stress (induced by hydroxyl radicals) in human plasma: protein carbonylation, thiol group concentration, and lipid peroxidation (determined by thiobarbituric acid reactive substances—TBARS). The fractions were also evaluated for procoagulant or anticoagulant activity by three hemostasis parameters of plasma: thrombin time (TT), prothrombin time (PT), and activated partial thromboplastin time (APTT).

## Materials and methods

### Chemicals

Methanol (isocratic grade), acetonitrile (LC–MS grade), formic acid (LC–MS grade), hexane, *n*-butanol, 5,5′-dithio-bis-(2-nitrobenzoic acid) (DTNB), guanidine hydrochloride, thiobarbituric acid (TBA), hydrogen peroxide, and sodium dodecyl sulfate (SDS) were from Merck (Darmstadt, Germany). Trichloroacetic acid (TCA), NaCl, EDTA, ethanol, and ethyl acetate were purchased from POCH (Gliwice, Poland). Serotonin was purchased from Thermofisher Scientific (Waltham, Massachusetts, US). All reagents needed for coagulation times assays were purchased from Diagon (Budapest, Hungary). All other reagents were purchased from commercial suppliers, including POCH (Gliwice, Poland), Merck (Darmstadt, Germany), and Chempur (Piekary Śląskie, Poland).

### Plant material

Sea buckthorn was obtained from a horticultural farm in Sokółka, Podlaskie Voivodship, Poland (53° 24′ N, 23° 30′ E), the largest plantation (33 ha) of this fruit in Poland. The plant material was identified by Mr. Stanisław Trzonkowski, the owner of the plantation. The ripe fruit was harvested in the second half of August, 2017. Whole branches were provided. A voucher specimen (IUNG/HRH/2017/1) was deposited at the Department of Biochemistry and Crop Quality, Institute of Soil Science and Plant Cultivation, State Research Institute, Puławy, Poland. The use of sea buckthorn in the present study complies with international, national and/or institutional guidelines.

The fruit was picked manually and frozen. To isolate the seeds, the thawed fruit was homogenized in water, using a blender. The fruit pulp and skins were washed out with tap water, and the sedimented seeds were collected, rinsed with methanol (to remove excess of water and facilitate drying), drained, and air-dried. The dry seeds were stored at room temperature.

A portion of sea buckthorn seeds was subjected to thermal treatment in a baking and pastry company, as described before^8^. The applied roasting conditions were chosen to mirror the process of rye bread baking. The temperature in the baking chamber at the beginning (approx. 10 min.) was 280 °C, the relative humidity was 35%. In the second phase, the temperature was decreased to 175 °C. The total roasting time was 36 min.

### Preparation of fractions a-g

The method of preparation of the extract from roasted seeds of sea buckthorn was described previously^8^. Briefly, the seeds were powdered in a coffee grinder, defatted by maceration in hexane at room temperature (30 min.), drained, and air-dried. The ultrasound-assisted extraction of the plant material (91 g) with 80% methanol (v/v) and pure methanol comprised 4 steps: 500 mL 80% methanol (30 min.), 400 mL 80% methanol (30 min.), 300 mL 80% methanol (30 min.), and 200 mL 100% methanol (20 min.). The extracts were filtered, pooled, concentrated in a rotary evaporator, and defatted by liquid–liquid extraction with hexane. The defatted extract was rotary-evaporated to remove all organic solvents and subjected to liquid–liquid extraction with butanol. Pooled butanol extracts were dried in a rotary-evaporator, dissolved in 20% *tert*-butanol, and lyophilized (Gamma 2–16 LSC, Christ, Osterode am Harz, Germany) to yield 1.93 g of the roasted seed extract.

To obtain fractions with distinct groups of compounds, the roasted seed extract was separated by reversed-phase liquid chromatography. A 1.04 g portion of the extract was shaken with 45 mL of 1.5% methanol with 0.1% formic acid (v/v), sonicated (3 min.), and centrifuged. The supernatant was loaded onto a short C18 open column (Cosmosil 140C18-PREP; self-packed, 66 mm × 44 mm), equilibrated with the same solvent. The column was washed with 300 ml of the solvent to remove highly polar compounds. The bound compounds were eluted sequentially with portions of 20%, 66%, and 85% methanol (300 mL each). The solubility of the extract in 1.5% methanol was poor. To separate all available extract, the pellet that remained after centrifugation was dissolved in 4.5 mL of methanol, and the solution was subsequently filled up with water to the volume of 45 mL, and acidified with 45 µL of formic acid. Then, it was centrifuged, and the obtained supernatant was loaded onto the same column, equilibrated with 10% methanol + 0.1% formic acid. The column was washed with the same solvent (200 mL), and the elution with 20%, 66% and 85% methanol was repeated, as described above. The pellet obtained after the second centrifugation was dissolved in 9 mL of methanol, and the solution was filled up to the volume of 45 mL and acidified with 45 µL of formic acid. The mixture was centrifuged, the supernatant was loaded onto the column equilibrated with 10% + 0.1% formic acid; the chromatographic separation was performed again. Corresponding eluates, obtained during the above-described chromatographic separations, were pooled, rotary evaporated to remove methanol, and freeze-dried, to yield 0.324 g of faction g (the 1.5% methanol washing), 0.106 g of fraction f (20% methanol eluates), 0.308 mg of fraction e (66% methanol eluates), and 0.147 g of fraction d (85% methanol eluates).

The extract from 52 g of raw seeds was prepared in a similar way (1.02 g). The fractionation of the extract was performed as described above, to yield 0.064 g of fraction c (20% methanol eluates), 0.223 g of fraction b (66% methanol eluates), and 0.121 g of fraction a (85% methanol eluates).

### Phytochemical analysis of the fractions a-g

The composition of the investigated fractions was analyzed by UHPLC-ESI-HRMS/MS, using a Thermo Ultimate 3000RS (Thermo Fischer Scientific, MS, USA) chromatographic system, equipped with a charged aerosol detector and coupled with a Bruker Impact II Q-TOF mass spectrometer (Bruker Daltonics GmbH, Bremen, Germany). Fraction g was dissolved in 1% methanol; fractions c and f—in 20% methanol; fractions b and e—in 50% methanol; fractions a and d—in 70% methanol. All samples had the same concentration (2.4 mg mL^−1^), except for fraction g, which was dissolved four times (to avoid unnecessarily contaminating the CAD detector and the ESI ions source with large amounts of unretained substances). The dissolved samples were centrifuged and analyzed. Chromatographic separations were performed at 40 °C, on an ACQUITY UPLC HSS C18 column (2.1 × 100 mm, 1.8 µm; Waters, MA, USA), equipped with a respective pre-column. The mobile phase A was 0.1% formic acid in MilliQ water, the mobile phase B was acetonitrile containing 0.1% of formic acid. The flow rate was 0.400 mL min^−1^. The injection volume was 3 µL. Fractions b, c, e, f, and g were chromatographed using the following elution program: from 0.0 to 1.0 min.—5% B, 1.0 to 26 min.—a concave gradient to 80% B; the column was subsequently washed with 99% B (26.5–28.0 min.), then the mobile phase returned to initial conditions (28.0–29.0 min.), and the column was equilibrated for 1 min. In the case of fractions a and d, the described elution method was slightly modified, the concave gradient was replaced by a linear one. HRMS analyses were performed using negative and positive ion modes. The following settings were applied in negative mode: the scanning range was *m/z* 80–2000; capillary voltage was 3 kV; dry gas flow was 10 L min^−1^; dry gas temperature was 220 °C; nebulizer pressure was 2.0 Bar; collision RF was 750 Vpp; transfer time was 100 µs; prepulse storage time was 10 µs. Collision energy was set automatically in the range from 2.5 to 80 eV, depending on the *m/z* values of fragmented ions. Settings for the positive ion mode: the scanning range was *m/z* 100–2000; capillary voltage was 4 kV; the remaining parameters were almost the same as those described above, but the automatically set collision energies ranged from 2.5 to 50 eV. The obtained data were calibrated internally with a solution of sodium formate, which was introduced to the ion source via a 20 μL loop at the beginning of each separation. However, when the above-described MS method was applied, triterpenoid saponins were ionized very weakly, and no fragmentation data was obtained for many of them. For this reason, the fraction d was subjected to another LC–MS analyses. The flow rate and the column temperature were as described above. The solvent A was 0.1% formic acid and 10 mM ammonium formate in MilliQ water, the solvent B was acetonitrile containing 0.1% formic acid. The injection volume was 2 µL. The elution method was as follows: from 0.0 to 1.0 min.—5% B; from 1.0 to 17.0 min.—a linear gradient to 90% B; from 17.0 to 20.5 min.—90% B; from 21.0 to 25.0 min.—5% B. HRMS analyses were performed in negative and positive ion mode. The following settings were applied in negative ion mode: the ion source settings were not modified; collision RF was 750 Vpp; transfer time was 120 µs; prepulse storage time was 10 µs. Collision energy was set automatically in the range from 20 to 60 eV, depending on the *m/z* values of fragmented ions. The settings for positive mode: the ion source settings were not modified; collision RF was 750 Vpp; transfer time was 120 µs; prepulse storage time was 10 µs. Collision energy was set automatically in the range from 15 to 40 eV. The use of the mobile phase with ammonia formate caused that saponins were effectively ionized in the positive ion mode, frequently forming adducts with two ammonia ions, and were fragmented by collision-induced dissociation. Samples e, and f were additionally analyzed using an ACQUITY UPLC chromatographic system (Waters MA, USA), equipped with a diode array detector (DAD) and a triple quadrupole mass detector (TQD), to obtain UV spectra of the separated substances, and facilitate their identification; samples were chromatographed on an ACQUITY BEH C18 column (2.1 × 100 mm; Waters, as described by Sławińska et al.^8^.

Constituents of the samples were tentatively identified on the basis of their MS spectra (with the help of Compound Crawler 3.3, a database search software (Bruker)), UV spectra (when possible), and literature data.

### Isolation of pure compounds

Isolation and structure determination of isorhamnetin 3-*O*-β-glucoside7-*O*-α-rhamnoside based on the UHPLC-MS analysis was described in Skalski et al.^14^.

### Preparation of stock solutions (fractions) and pure compounds for bioassays

The fractions were dissolved in 50% DMSO (a universal solvent for many different plant substances) and the pure compounds in 75% DMSO. The final concentration of DMSO in the tested human plasma was below 0.5% (*v*/*v*) (for the fractions) and 0.75% (*v/v*) (for the compounds). The addition of a low concentration of DMSO to human plasma has no effect on its hemostatic properties and the level of oxidative stress (data not presented).

### Blood samples

Human whole blood was collected from “Diagnostyka” blood collection center at Brzechwy 7A (Łodź, Poland). All donors signed an informed consent form one day before blood collection. The volunteers did not smoke, drink alcohol, or take medication (including antioxidant or antiplatelet supplements or drugs) for two weeks prior. Blood was drawn into tubes with CPDA anticoagulant (citrate/phosphate/dextrose/adenine; 8.5:1; *v*/*v*; blood/CPDA). Plasma was isolated from full blood by differential centrifugation (2800 × g, 20 min, room temperature). The research was conducted according to the guidelines of the Helsinki Declaration for Human Research, with the approval of Bioethics Committee at the University of Łódź (11/KBBN-UŁ/I/2019).

### Lipid peroxidation measurement

Lipid peroxidation was measured based on the concentration of thiobarbituric acid-reactive substances (TBARS) as described earlier by Sławińska et al.^8^. Plasma was incubated with fractions or compounds and an oxidative stress inducer (4.7 mM H_2_O_2_/3.8 mM Fe^2+^/2.5 mM EDTA) for 30 min at 37 °C (final concentrations: 1, 10, and 50 μg/mL). A negative control with 0.9% NaCl was set up. After the incubation, equal amounts of 15% TCA in 0.25 M HCl and 0.37% TBA in 0.25 M HCl were added to the samples; the samples were mixed and placed in a 100 °C thermoblock for 15 min. Next, the samples were cooled and centrifuged (10 000 × g, 15 min, 18 °C). The absorbance of the supernatant was measured in triplicate with a SPECTROstar Nano Microplate Reader (BMG LABTECH, Germany) at 535 nm. Equal amounts of 0.9% NaCl, 15% TCA, and 0.37% TBA were used as a blank sample. TBARS concentration was calculated with a molar extinction coefficient (ε = 156,000 M^−1^ cm^−1^).

### Protein carbonylation measurement

Protein carbonylation was measured with a method employing 2,4-dinitrophenylhydrazine (DNPH) as described earlier by Sławińska et al.^8^. Plasma was incubated with the fractions or compounds and an oxidative stress inducer (4.7 mM H_2_O_2_/3.8 mM Fe^2+^/2.5 mM EDTA) for 30 min at 37 °C (final concentrations: 1, 10, and 50 μg/mL). After the incubation, the samples were diluted 10 times with 0.9% NaCl. Then, 40% TCA was added on ice. The samples were incubated for 5 min and centrifuged (5 min, 2500 rpm, 4 °C). Supernatant was discarded and 10 mM DNPH in 2 M HCl was added to the pellets. The samples were incubated for 1 h at room temperature, in the dark. 40% TCA was added on ice, the samples were incubated for 5 min and centrifuged again (5 min, 2500 rpm, 4 °C). The supernatant was discarded. 1.5 mL of 1:1 ethanol/ethyl acetate was added to the pellets on ice and the samples were vortexed for 5 min and centrifuged (5 min, 2500 rpm, 4 °C); this step was repeated two more times. Finally, supernatant was discarded, and the pellets were dissolved in 1 mL 6 M guanidine hydrochloride in 2 M HCl. The absorbance was measured in triplicate with a SPECTROstar Nano Microplate Reader (BMG LABTECH, Germany) at 280 and 375 nm. The levels of carbonyl groups were calculated with a molar extinction coefficient (ε = 22,000 M^−1^ cm^−1^) and expressed as nmol carbonyl groups/mg of plasma protein.

### Thiol group oxidation measurement

The levels of thiol groups were measured with a method based on Ellman’s reagent (5,5′-dithio-bis-(2-nitrobenzoic acid), DTNB) as described earlier by Sławińska et al.^8^. Plasma was incubated with fractions or compounds and an oxidative stress inducer (4.7 mM H_2_O_2_/3.8 mM Fe^2+^/2.5 mM EDTA) for 30 min at 37 °C (final concentrations: 1, 10, and 50 μg/mL). A negative control with 0.9% NaCl was set up. After the incubation 20 μL of the samples were added to a 96-well plate in triplicate. 20 μL of 10% SDS in 10 mM phosphate buffer (pH 8) and 160 μL of 10 mM phosphate buffer (pH 8) were added to all samples. Absorbance was measured with a SPECTROstar Nano Microplate Reader (BMG LABTECH, Germany) at 412 nm and 280 nm. Afterward, 16.6 μL of 10 mM DTNB in 10 mM phosphate buffer (pH 8) was added to the samples (16.6 μL of 10 mM phosphate buffer (pH 8) was added to the blank sample instead of DTNB). The plate was covered with parafilm and incubated at 37 °C for 1 h. Afterward, the absorbance was read again. The levels of thiol groups were calculated with a molar extinction coefficient (ε = 13,600 M^−1^ cm^−1^) and expressed as nmol carbonyl groups/mg of plasma protein.

### Measurement of prothrombin time

Plasma was incubated with the fractions for 30 min at 37 °C; the final concentrations were 10 and 50 μg/mL. A control sample with 0.9% NaCl was set up. 50 μL of the samples were added to coagulometer cuvettes in duplicate. The samples were incubated again for 2 min at 37 °C and 100 μL of Dia-PT reagent was added. The time of coagulation was recorded with a K-3002 Optic Coagulometer (Kselmed) as described earlier by Sławińska et al.^8^.

### Measurement of thrombin time

Plasma was incubated with the fractions for 30 min at 37 °C; the final concentrations were 10 and 50 μg/mL. A control sample with 0.9% NaCl was set up. 50 μL of the samples were added to coagulometer cuvettes in duplicate. The samples were incubated for 1 min at 37 °C and 100 μL of thrombin (final concentration – 5 U/mL) was added. The time of coagulation was recorded with a K-3002 Optic Coagulometer (Kselmed) as described earlier by Sławińska et al.^8^.

### Measurement of activated partial thromboplastin time

Plasma was incubated with the fractions for 30 min at 37 °C; the final concentrations were 10 and 50 μg/mL. A control sample with 0.9% NaCl was set up. 50 μL of the samples were added to coagulometer cuvettes in duplicate. 50 μL of Dia-PTT reagent (pre-heated to 37 °C) was added and the samples were incubated for 3 min at 37 °C. Afterwards, 50 μL of Dia-CaCl_2_ reagent was added. The time of coagulation was recorded with a K-3002 Optic Coagulometer (Kselmed) as described earlier by Sławińska et al.^8^.

### Statistical analysis

Statistical analysis was carried out with Statistica 10 (StatSoft 13.3, TIBCO Software Inc. Palo Alto, CA, USA). The distribution of data was checked with Shapiro–Wilk test. The homogeneity of variance was checked with Levene’s test. If the data had normal distribution and homogenous variance, one-way ANOVA with Tukey’s post-hoc test was used to assess the differences between groups; otherwise, Kruskal–Wallis test was used. The results are presented as means ± SD or medians and interquartile ranges. The results were considered significant at p < 0.05. Dixon’s Q-test was used to eliminate uncertain data.

## Results

### Chemical characteristic of fraction a-g

**Fig. 1:**
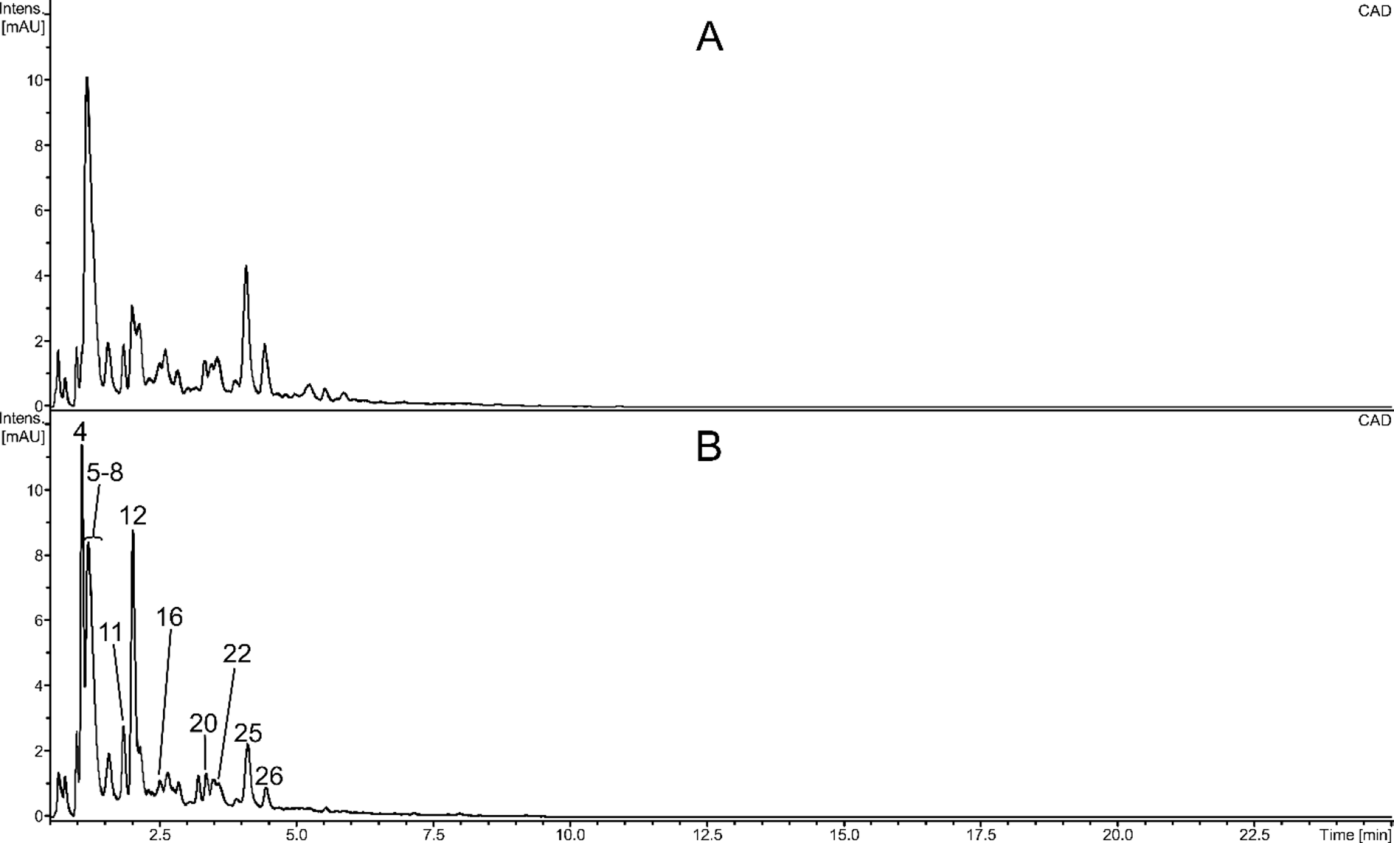
UHPLC-CAD chromatograms of fractions c (**A**) and f (**B**). Numbers above the chromatographic peaks correspond to numbers of compounds in Table 1.

Fraction g consisted of the most polar compounds, mainly dihexoses. It also contained small amounts of trihexoses, as well as putative dipeptide and serotonin. The composition of fractions c and f (eluted with 20% methanol) was similar, but they differed markedly in the content of some compounds. Serotonin seemed to be the dominant compound of both preparations. Serotonin hexoside was also detected. A sulfur-containing putative dipeptide and a putative indolylacetic acid hexoside were the other major constituents of fraction f. They were also present in fraction c but in significantly lower amounts (Fig. 1, Table 1). Moreover, both fractions contained putative nucleosides (uridine, guanosine, xanthosine), aromatic amino acids (phenylalanine, tryptophan), methylgallic acid, and diverse B-type proanthocyanidins (dimers, and trimers of (epi)catechin, and/or (epi)gallocatechin). Procyanidin peaks of fraction c were about two times higher than those of fraction f (Fig. 1).

**Table 1:** Tentatively identified constituents of fractions c and f.

No	tR	Precursor ion	*m/z*	CID	Error (ppm)	mσ	Formula	Tentative identification
1	0.71	[M + FA-H]^−^	387.1145	341.1092 (100). 179.0573 (14)	− 0.2	16.7	C_12_H_22_O_11_	dihexose
2	0.99	[M-H]^−^	243.0622	243.0624 (67), 200.0566 (100), 182.0461 (2), 152.0353 (14), 140.0341 (8), 110.0240 (9)	0.2	9.6	C_9_H_12_N_2_O_6_	Uridine / isomer
3	0.99	[M + FA-H]^−^	312.0951	266.0894 (57), 134.0466 (100)	− 0.5	7.8	C_10_H_13_N_5_O_4_	Adenosine / isomer
4	1.1	[M-H]^−^	263.0704	263.0705 (18), 245.0605 (8), 227.0493 (5), 215.0675 (7), 197.0569 (2), 179.0462 (3), 171.0779 (100), 153.0666 (12), 128.0353 (57)	1.3	5.3	C_9_H_16_N_2_O_5_S	Putative dipeptide
		[M + H]^+^	265.0851	265.0852 (100), 248.0586 (9), 202.0533 (4), 136.0426 (9), 119.0160 (7)	0.5	10.8		
5	1.18	[M + H]^+^	177.1021	177.1022 (12), 160.0757 (100)	1.0	4.4	C_10_H_12_N_2_O	Serotonin / isomer
6	1.21	[M-H]^−^	337.1408	337.1400. (58), 247.1079 (30), 241.0990 (100), 199.0876 (74), 187.0883 (34), 175.0869 (15)	− 1.0	6.4	C_16_H_22_N_2_O_6_	Serotonin hexoside
7	1.21	[M-H]^−^	283.0682	283.0682 (80), 151.0256 (100)	0.8	14.4	C_10_H_12_N_4_O_6_	Xanthosine / isomer
8	1.26	[M-H]^−^	183.0299	183.03 (100), 165.0184 (2), 139.0394 (11)	− 2.0	6.3	C_8_H_8_O_5_	Methylgallic acid
9	1.53	[M-H]^−^	609.1259	441.0833 (2), 423.0724 (100), 355.0826 (8), 305.0669 (65), 297.0407 (8), 283.0252 (10), 273.0405 (7), 261.0765 (7), 177.0196 (20), 125.0249 (5)	− 1.5	12.4	C_30_H_26_O_14_	(epi)GC-(epi)GC
10	1.59	[M-H]^−^	303.0989	259.1087 (100), 241.0983 (20), 215.1189 (77), 186.0921 (3)	− 0.8	5.8	C_15_H_16_N_2_O_5_	Unidentified
11	1.86	[M-H]^−^	164.0714	164.0719 (100), 147.0448 (44)	1.6	16.9	C_9_H_11_NO_2_	Phenylalanine
12	1.97	[M-H]^−^	368.0986	368.0989 (3), 188.0356 (12), 179.0567 (2), 144.0450 (100), 119.0354 (3), 113.0251 (3)	− 1.3	1.8	C_16_H_19_NO_9_	Unidentified hexoside (indoleacetic acid derivative ?)
		[M + H]^+^	370.1124	208.0600 (72), 190.0494 (100), 146.0596 (34)	2.4	5.8		
13	2.08	[M + H]^+^	465.1648	465.1648 (45), 448.1382 (100), 289.0700 (97), 177.1017 (23), 160.0753 (11)	1.9	12.6	C_25_H_24_N_2_O_7_	Unidentified (serotonin derivative?)
14	2.13	[M-H]^−^	305.0672	305.0673 (100), 287.0566 (4), 261.0774 (33), 237.0774 (12), 233.0823 (3), 221.0459 (27), 219.0667 (48), 179.0356 (24), 167.0353 (20), 165.0195 (20), 125.0251 (23)	− 1.6	3.8	C_15_H_14_O_7_	(epi)GC
15	2.29	[M-H]^−^	218.1034	218.1035 (100), 146.0817 (26)	− 0.2	3.6	C_9_H_17_NO_5_	Pantothenic acid / isomer
16	2.51	[M-H]^−^	609.1253	441.0823 (3), 423.0724 (100), 355.0823 (9), 305.0668 (83), 297.0406 (8), 283.0249 (9), 273.0406 (9), 261.0767 (8), 177.0196 (25), 125.0250 (5)	− 0.4	8.5	C_30_H_26_O_14_	(epi)GC-(epi)GC
17	2.70	[M + H]^+^	465.1649	465.1648 (39), 448.1385 (100), 430.1276 (8), 338.1017 (7), 289.0701 (77), 177.1016 (18), 160.0752 (9)	1.6	10.3	C_25_H_24_N_2_O_7_	Unidentified(serotonin derivative ?)
18	2.86	[M-H]^−^	593.1298	467.0997 (2), 423.0724 (100), 355.0828 (10), 305.067 (88), 297.0409 (8), 289.072 (9), 283.025 (10), 273.0406 (6), 261.0769 (9), 219.0662 (6), 125.0251 (6)	0.5	8.7	C_30_H_26_O_13_	(epi)GC-(epi)C
19	3.20	[M-H]^−^	396.0938	188.0346 (12), 144.0446 (100), 113.0265 (2)			C_17_H_19_NO_10_	Unidentified
20	3.32	[M-H]^−^	203.0823	203.0828 (100), 186.0560 (3), 159.0925 (6), 142.0658 (3), 116.0513 (11)	− 2.2	3.3	C_11_H_12_N_2_O_2_	Tryptophan
21	3.43	[M + H]^+^	465.1644	465.1643 (53), 448.1382 (99), 430.1274 (14), 418.1271 (13), 338.1013 (12), 289.0697 (100), 177.1014 (25), 160.0750 (12)	2.6	8.4	C_25_H_24_N_2_O_7_	Unidentified (serotonin derivative?)
22	3.59	[M-H]^−^	593.1305	467.0989 (2), 423.073 (100), 355.0832 (9), 305.0673 (90), 299.0563 (2), 297.041 (8), 289.0723 (8), 287.0563 (5), 283.0252 (9), 261.0771 (9),125.0251 (7)	− 1.4	4.7	C_30_H_26_O_13_	(epi)GC-(epi)C
23	3.92	[M-H]^−^	305.0663	305.0669 (100), 287.0561 (6), 261.0769 (33), 237.0769 (11), 233.0818 (3), 221.0455 (28), 219.0664 (50), 179.0355 (25), 167.0349 (20), 165.0194 (18), 125.0249 (22)	− 0.9	10.3	C_15_H_14_O_7_	(epi)GC
24	3.92	[M-H]^−^	577.1359	451.1033 (2), 425.0889 (5), 407.0784 (69), 381.0989 (5), 339.0884 (12), 289.0726 (100), 273.0409 (6), 245.0825 (22), 125.0255 (6)	− 1.3	27.1	C_30_H_26_O_12_	(epi)C-(epi)C
25	4.09	[M-H]^−^	577.1353	451.1046 (2), 425.0885 (6), 407.0778 (93), 381.0990 (6), 339.0881 (16), 289.0723 (100), 273.0411 (9), 245.0824 (21), 125.0251 (7)	− 0.3	12.4	C_30_H_26_O_12_	(epi)C-(epi)C
26	4.42	[M-H]^−^	865.1991	577.1357 (2), 543.0947 (6),525.084 (12), 451.1043 (9), 449.0888 (8), 425.0884 (9), 407.0781 (79), 391.0468 (6), 341.0674 (9), 289.0725 (100), 273.0409 (8), 261.0409 (31), 245.0458 (20), 243.0304 (35), 125.0249 (13)	− 0.7	3.4	C_45_H_38_O_18_	(epi)C-(epi)C-(epi)C
27	5.25	[M-H]^−^	577.1359	451.1044 (2), 425.0883 (5), 407.0781 (83), 381.0988 (6), 339.088 (15), 289.0723 (100), 273.041 (8), 245.0824 (22), 125.0254 (6)	− 1.2	7.6	C_30_H_26_O_12_	(epi)C-(epi)C

(epi)C – (epi)catechin; (epi)GC – (epi)callocatechin.

**Fig. 2:**
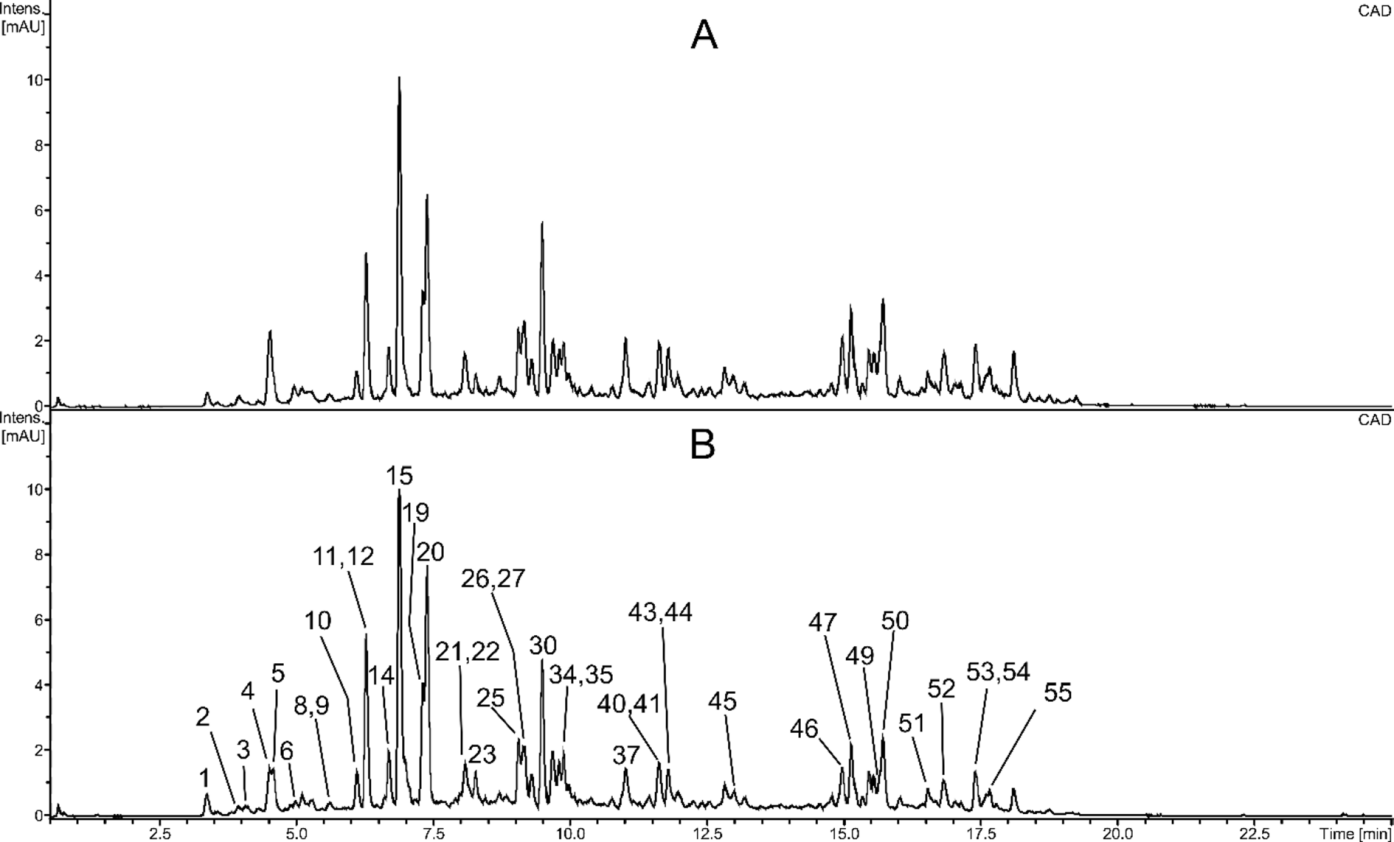
UHPLC-CAD chromatograms of fractions b (**A**) and e (**B**). Numbers above the chromatographic peaks correspond to numbers of compounds in Table 2.

Fractions b and e (eluted with 66% methanol) were almost identical and consisted mainly of diverse flavonol glycosides (Fig. 2, Table 2). Among them, 3-*O*-dihexoside-7-*O*-deoxyhexosides of kaempferol, isorhamnetin, and quercetin were the dominant compounds; isorhamnetin 3-*O*-glucoside-7-*O*-rhamnoside (confirmed using the authentic standard) was another major constituent. In addition to numerous simple flavonol glycosides, the fractions also contained many compounds acylated with phenolic acids (sinapic, ferulic acid), (2*E*)-2,6-dimethyl-6-hydroxy-2,7-octadienolic acid ((*E*)-linalool-1-oic acid), or both of them, occurring usually in small amounts. Peaks of acylated flavonoids in fraction e tended to be lower than those of fraction b. In addition, fractions b and e contained dimeric proanthocyanidins, catechin, epicatechin, (epi)gallicatechin, phenolic acid derivatives, alkaloids (such as tentatively identified harmalol and harmol), sesquiterpenoid hexosides, a vomifoliol glycoside, and several triterpenoid saponins. Most of these compounds occurred in small amounts.

**Table 2:** Tentatively identified constituents of fractions b and e.

No	tR	Precursor ion	*m/z*	CID	Error (ppm)	mσ	Formula	Tentative identification
1	3.38	[M-H]^−^	203.0826	203.0828 (100), 186.0564 (5), 159.0933 (6), 142.0648 (4), 116.0510 (10)	− 0.1	14.6	C_11_H_12_N_2_O_2_	Tryptophan
2	3.94	[M-H]^−^	305.0664	305.0669 (100), 287.0561 (6), 261.0769 (33), 237.0769 (11), 233.0818 (3), 221.0455 (28), 219.0664 (50), 179.0355 (25), 167.0349 (20), 165.0194 (18), 125.0249 (22	1.0	6.9	C_15_H_14_O_7_	(epi)gallocatechin
3	4.10	[M-H]^−^	577.1341	451.1053 (3), 425.0855 (6), 407.0764 (98), 381.0962 (9), 339.0868 (15), 289.0705 (100), 273.0386 (8), 245.0800 (28), 205.0498 (6), 125.0235 (8)	1.9	5.7	C_30_H_26_O_12_	(epi)C-(epi)C
4	4.51	[M-H]^−^	289.0719	289.0720 (100), 271.061 (3), 245.082 (75), 227.0717 (6), 221.0822 (14), 205.0508 (18), 203.0715 (22), 125.0252 (3)	− 0.4	4.4	C_15_H_14_O_6_	Catechin
5	4.60	[M + H + NH_3_]^+^	462.2326	427.1954 (4), 265.1427 (100), 247.1321 (11)	1.6	18.6	C_21_H_32_O_10_	Sesquiterpenoid hexoside
6	4.96	[M-H]^−^	199.0877	199.0865 (100), 184.0638 (2), 172. 0763 (1), 158.0596 (2)	− 0.2	6.9	C_12_H_12_N_2_O	Harmalol / isomer
7	5.28	[M-H]^−^	577.1359	451.1037 (3), 425.0885 (5), 407.0779 (85), 381.0991 (7), 339.0880 (16), 289.0721 (100), 273.0411 (8), 245.0822 (25), 205.0509 (5), 125.0246 (7)	− 1.2	11.0	C_30_H_26_O_12_	(epi)C-(epi)C
8	5.58	[M + H]^+^	199.0860	199.0859 (100)	3.0	1.9	C_12_H_10_N_2_O	Harmol / isomer
9	5.59	[M-H]^−^	255.0777	255.0776 (100), 237.0672 (91), 211.0879 (91), 209.0719 (10), 196.0658 (2)	− 0.6	1.8	C_14_H_12_N_2_O_3_	Unidentified alkaloid
10	6.00	[M-H]^−^	771.1992	771.1997 (7), 625.1389 (16), 446.0834 (100), 300.0249 (16), 299.0183 (39)	− 0.3	17.3	C_33_H_40_O_21_	Q-3-O-Hex_2_-7-O-dHex
11	6.29	[M-H]^−^	771.1992	771.1997 (87), 625.1389 (17), 446.0860 (100), 300.025 (18), 299.0183 (41)	− 0.4	4.2	C_33_H_40_O_21_	Q-3-O-Hex_2_-7-O-dHex
12	6.29	[M-H]^−^	289.0723	289.0725 (100), 271.0615 (3), 245.0830 (67), 227.0704 (4), 221.0801 (13), 205.0512 (20), 203.0701 (20), 179.0336 (8), 125.0238 (3)	− 2.0	12.3	C_15_H_14_O_6_	Epicatechin
13	6.34	[M-H]^−^	337.0930	290.0758 (7), 191.0567 (100), 173.0466 (8), 163.0399 (4)	− 0.4	23.6	C_16_H_18_O_8_	Coumaroylquinic acid
14	6.67	[M-H]^−^	755.2051	609.1474 (100), 430.0914 (86), 285.0407 (90), 255.0307 (3)	− 1.4	9.8	C_33_H_40_O_21_	K-3-O-Hex_2_-7-O-dHex
15	6.89	[M-H]^−^	755.2048	609.147 (100), 430.0912 (74), 284.033 (82), 255.0305 (3)	− 1.1	2.0	C_33_H_40_O_21_	K-3-O-Hex_2_-7-O-dHex
16	7.00	[M + FA-H]^−^	563.2328	517.2275 (100), 385.1856 (8), 365.1447 (5), 293.0868 (19), 233.0657 (26), 223.1323 (4), 205.1223 (30), 191.055 (14), 149.044 (7)	3.0	2.0	C_24_H_38_O_12_	Vomifoliol-Hex-Pen
17	7.24	[M-H]^−^	771.2001	771.1998 (15), 591.1370 (2), 300.0282 (100)	− 1.5	8.7	C_33_H_40_O_21_	Q-3-O-dHex-Hex_2_
18	7.30	[M-H]^−^	234.1137	234.1137 (100), 119.0507 (11)	− 0.5	3.7	C_13_H_17_NO_3_	Coumaric acid derivative
		[M + H]^+^	236.1273	236.1272 (100), 147.0434 (42)	3.5	8.4		
19	7.30	[M-H]^−^	785.2141	639.1575 (72), 460.1019 (49), 315.0512 (100)	0.6	6.4	C_34_H_42_O_21_	I-3-O-Hex_2_-7-O-dHex
20	7.41	[M-H]^−^	785.2154	639.1577 (100), 460.1019 (27), 314.0439 (36)	− 1.0	6.4	C_34_H_42_O_21_	I-3-O-Hex_2_-7-O-dHex
21	8.04	[M-H]^−^	609.1475	609.1471 (15), 463.0892 (13), 446.0863 (100), 300.0257 (9), 299.0205 (45)	− 1.3	1.3	C_27_H_30_O_16_	Q-3-O-Hex-7-O-dHex
22	8.10	[M-H]^−^	755.2056	755.2051 (13), 300.0284 (100), 271.0258 (3)	− 2.2	13.9	C_33_H_40_O_20_	Q-3-O-dHex_2_-Hex
23	8.29	[M + FA-H]^−^	461.1672	415.1591 (100), 311.0969 (3), 293.0866 (5), 251.0757 (34), 233.0657 (5), 221.0656 (13), 191.055 (84), 179.0548 (8), 161.0453 (4), 149.0439 (50), 131.0333 (8)	− 1.7	7.0	C_19_H_28_O_10_	Unidentified glycoside
24	8.71	[M-H]^−^	319.0832	319.0833 (16), 163.0407 (100), 155.0349 (10), 145.0296 (7), 137.0246 (14), 119.051 (19)	− 2.6	6.3	C_16_H_16_O_7_	Coumaroylshikimic acid
25	9.04	[M-H]^−^	739.2099	739.2099 (20), 593.1524 (21), 430.0916 (7), 284.0333 (100), 255.0306 (6)	− 1.1	12.6	C_33_H_40_O_19_	K-3-O-Hex-dHex-7-O-dHex
26	9.18	[M-H]^−^	593.1524	593.1524 (10), 447.0945 (24), 430.0914 (100), 285.0411 (63), 283.0255 (55)	− 2.0	15.2	C_27_H_30_O_15_	K-3-O-Hex-7-O-dHex
27	9.18	[M-H]^−^	623.1628	477.1052 (85), 460.1024 (100), 315.0518 (80)	− 1.7	4.7	C_28_H_32_O_16_	I-3-O-Hex-7-O-dHex
28	9.31	[M-H]^−^	609.1471	609.1477 (28), 300.0286 (100)	− 1.6	13.6	C_27_H_30_O_16_	Q-3-O-Hex-dHex (rutin?)
29	9.31	[M-H]^−^	769.2211	769.2209 (17), 623.1631 (16), 605.1527 (2), 314.0440 (100)	− 1.9	6.9	C_34_H_42_O_20_	I-3-O-Hex-dHex-7-O-dHex
30	9.50	[M-H]^−^	623.1628	623.1631 (6), 477.1052 (26), 460.1022 (100), 315.0520 (77)	− 1.7	2.6	C_28_H_32_O_16_	I-3-O-Hex-7-O-dHex
31	9.69	[M-H]^−^	441.1772	441.1776 (80), 397.1875 (33), 330.1328 (100), 261.1141 (8), 235.1349 (7), 217.1238 (17), 205.1236 (13)	− 1.3	4.8	C_21_H_30_O_10_	Sesquiterpenoid glycoside
32	9.69	[M-H]^−^	961.2625	815.2051 (44), 609.1474 (39), 430.0915 (43), 284.0333 (100)	− 0.6	4.0	C_44_H_50_O_24_	K-3-O-Hex_2_-7-O-dHex-SinA
33	9.80	[M-H]^−^	991.2733	845.2153 (30), 639.1578 (20), 460.1017 (24), 314.0438 (100)	− 0.8	8.7	C_45_H_52_O_25_	I-3-O-Hex_2_-7-O-dHex-SinA
34	9.91	[M-H]^−^	403.1617	223.0890 (100), 205.0879 (2), 179.1086 (45)	− 1.7	13.0	C_18_H_28_O_10_	Unidentified glycoside
35	9.91	[M-H]^−^	933.2518	785.1947 (40), 609.1472 (35), 430.0913 (32), 284.0333 (100)	− 0.5	13.8	C_43_H_48_O_23_	K-3-O-Hex_2_-7-O-dHex-FerA
36	10.77	[M-H]^−^	623.1627	623.1632 (37), 314.0440 (100), 299.0207 (16)	− 1.5	13.5	C_28_H_32_O_16_	I-3-O-Hex-dHex
37	11.01	[M-H]^−^	623.1628	623.1632 (16), 315.0515 (100)	− 1.6	11.5	C_28_H_32_O_16_	I-3-O-Hex-dHex
38	11.01	[M-H]^−^	855.2572	709.2003 (13), 446.0870 (100), 299.0208 (46)	− 0.9	17.2	C_38_H_48_O_22_	Q-3-O-Hex-dHex-IvaA-7-O-dHex
39	11.44	[M + H]^+^	479.1180	317.0655 (100)	0.8	11.5	C_22_H_22_O_12_	I-3-O-Hex
40	11.63	[M-H]^−^	187.0983	187.0985 (100), 169.0878 (14), 125.0980 (24)	− 4.1	0.5	C_9_H_16_O_4_	Unidentified aliphatic acid
41	11.63	[M-H]^−^	481.1554	211.0444 (34), 193.0323 (6), 181.0335 (4), 120.9971 (100)				Unidentified
42	11.63	[M + H]^+^	923.3163	595.1651 (5), 433.1125 (100), 311.1490 (9), 287.0548 (51)	1.8	12.9	C_43_H_54_O_22_	K-3-O-Hex_2_-LinA-7-O-dHex
43	11.80	[M-H]^−^	839.2621	693.2048 (80), 609.1477 (5), 430.0914 (97), 284.0331 (100)	− 0.7	23.2	C_38_H_48_O_21_	K-3-O-Hex_2_-IvaA-7-O-dHex
44	11.80	[M-H]^−^	951.3145	805.2575 (61), 639.1580 (6), 460.1022 (50), 314.0440 (100)	− 0.5	15.0	C_44_H_56_O_23_	I-3-O-Hex_2_-LinA-7-O-dHex
45	12.97	[M-H]^−^	1105.5401	1105.5436 (100), 943.4915 (9), 925.4805 (26), 781.4377 (9), 687.4116 (11), 619.3858 (70), 601.7350 (20), 487.3439 (4)	1.8	85.2	C_53_H_86_O_24_	C_30_H_48_O_5_-3Hex-Pen
46	14.98	[M + H]^+^	939.3118	777.2593 (12), 615.2072 (57), 597.1966 (45), 579.1863 (18), 451.1390 (9), 313.1650 (72), 303.0503 (100), 295.1543 (22)	1.2	7.4	C_43_H_54_O_23_	Q-3-O-Hex_2_-7-O-dHex-LinA
47	15.13	[M-H]^−^	1293.6083	1293.6083 (67), 1147.5515 (10), 1131.5559 (95), 1113.5453 (55), 985.4992 (13)969.5039 (11), 807.4516 (100), 789.4409 (11), 661.3938 (17, 643.3839 (11), 529.3532 (16), 469.3316 (2)	2.9	20.9	C_61_H_98_O_29_	Triterpenoid saponin
48	15.55	[M-H]^−^	921.3015	775.2445 (2), 609.1458 m(57), 596.1893 (100), 429.0824 (9), 283.0243 (82)	2.1	14.0	C_43_H_54_O_22_	K-3-O-Hex_2_-7-O-dHex-LinA
49	15.65	[M-H]^−^	1147.5521	1147.5518 (95), 1087.5321 (8), 985.5001 (100), 967.4895 (34), 823.4475 (11), 661.3952 (84), 643.3842 (29), 619.3836 (4), 601.3744 (11), 529.3534 (6)	1.8	42.0	C_55_H_88_O_25_	C_30_H_48_O_5_-Hex_3_-Pen-AcA
50	15.72	[M + H]^+^	953.3272	791.2747 (8), 629.2225 (60), 611.2121 (36), 593.2016 (14), 465.1544 (7), 317.0659 (100), 313.1648 (62), 295.1541 (19)	1.4	7.0	C_44_H_56_O_23_	I-3-O-Hex_2_-7-O-dHex-LinA
51	16.54	[M + 2FA-2H]^2−^	744.3316	1073.5507 (7), 911.4989 (15), 749.4470 (35), 603.3893 (5), 536.2729 (83), 471.3482 (2), 455.2467 (100), 374.2202 (44)	− 0.5	33.4	C_65_H_106_O_32_	C_30_H_48_O_4_-Pen-dHex-Hex_4_
52	16.84	[M + H]^+^	1145.3690	777.2597 (6), 615.2068 (67), 697.1963 (52), 579.1858 (21), 451.1384 (11), 369.1180 (31), 313.1647 (83), 303.0500 (100), 295.1538 (25), 207.0651 (65)	1.5	15.1	C_54_H_64_O_27_	Q-3-O-Hex_2_-SinA-7-O-dHex-LinA
53	17.43	[M-H]^−^	1127.3622	921.3053 (2), 815.2045 (37), 623.1604 (4), 609.147 (36), 596.1902 (92), 429.0821 (13), 283.0247 (100)	− 0.8	27.8	C_54_H_64_O_26_	K-3-O-Hex_2_-SinA-7-O-dHex-LinA
54	17.43	[M + H]^+^	1159.3839	791.2746 (4), 629.2221 (55), 611.2117 (36), 593.2011 (14), 465.1540 (7), 369.1179 (22), 317.0656 (100), 313.1645 (60), 295.1538 (18), 207.0649 (66)	2.2	19.8	C_55_H_66_O_27_	I-3-O-Hex_2_-SinA-7-O-dHex-LinA
55	17.68	[M-H]^−^	789.2611	626.2003 (57), 611.1770 (8), 477.1039 (20), 315.0509 (100)	0.1	6.0	C_38_H_46_O_18_	I-3-O-Hex-7-O-dHex-LinA
56	18.18	[M-H]^−^	327.2125	327.2171 (100), 309.2073 (3), 291.1956 (6), 239.1283 (4), 229.1440 (18), 211.1335 (18), 171.1025 (5)	0.6	12.4	C_18_H_32_O_5_	Unidentified fatty acid / isomer

*FA* formic acid; *I* isorhamnetin, *K* kaempferol; *Q* quercetin; *Hex* hexose; *dHex* deoxyhexose; *Pen* pentose; *AcA* acetic acid; *FerA* ferulic acid; *IvaA* isovaleric acid; LinA – (E)-linalool-1-oic acid; SinA – sinapic acid; (epi)C – (epi)catechin.

**Fig. 3:**
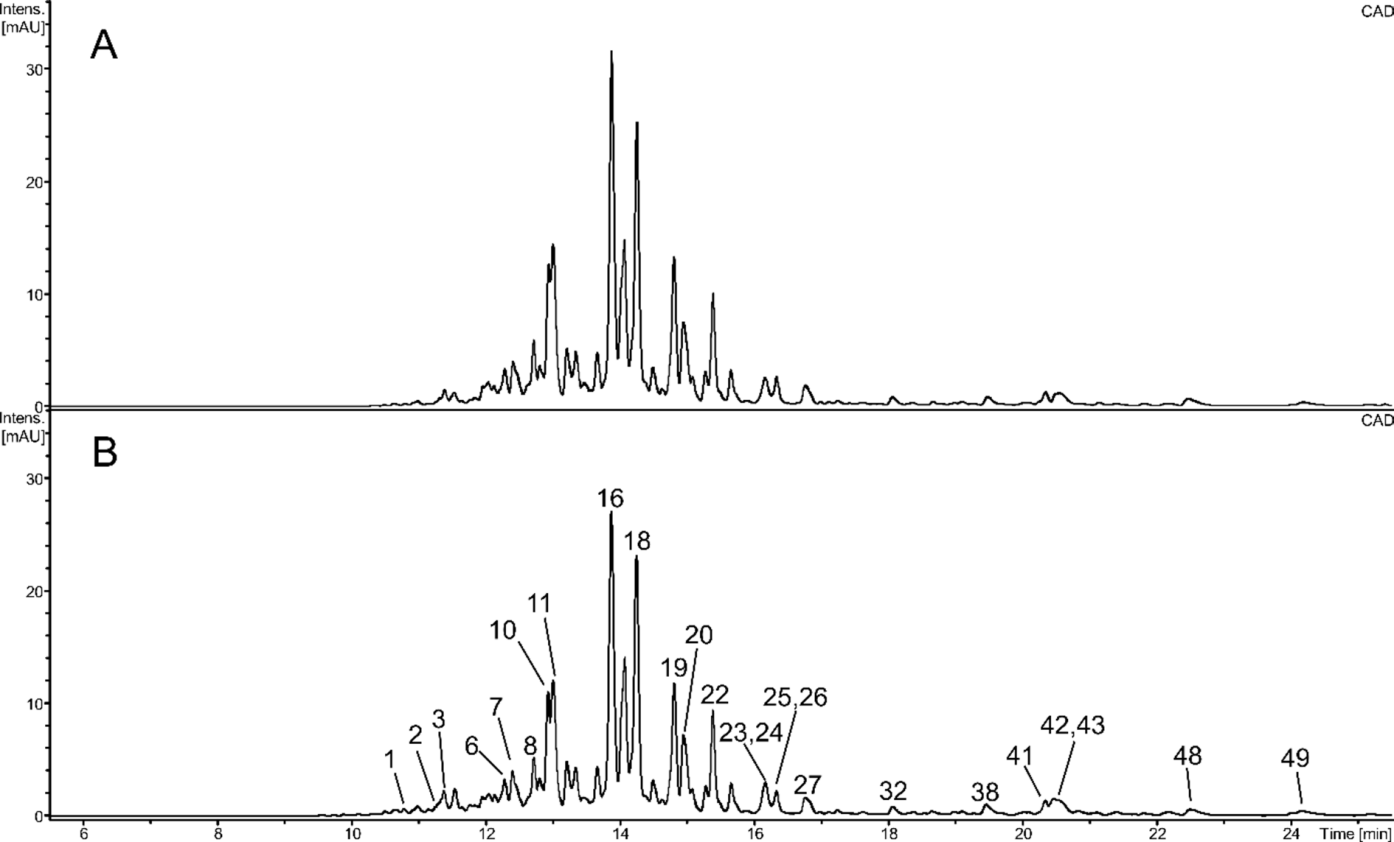
UHPLC-CAD chromatograms of fractions a (**A**) and d (**B**). Numbers above the chromatographic peaks correspond to numbers of compounds in Table 3.

Fractions a and d were virtually the same. They consisted mainly of many triterpenoid saponins; most of them were acylated with (2*E*)-2,6-dimethyl-6-hydroxy-2,7-octadienolic acid (Fig. 3, Table 3). The fractions also contained triterpenoids, putative hydroxylated fatty acids, diverse lysophospholipids, glycerophospho N-acylethanolamines, and small amounts of flavonoids acylated with (2*E*)-2,6-dimethyl-6-hydroxy-2,7-octadienolic acid.

**Table 3:** Tentatively identified constituents of fractions a and d.

No	tR	Precursor ion	*m/z*	CID	Error (ppm)	mσ	Formula	Tentative identification
1	10.78	[M + 2FA-2H]^2−^	663.3072	1073.555 (100), 911.5026 (95), 893.49.13 (50), 749.4494 (81), 687.4492 (17), 603.3920 (11), 536.2730 (13), 471.3489 (10), 455.2477 (20)	− 3.0	40.7	C_59_H_96_O_27_	C_30_H_48_O_4_-Pen-dHex-Hex_3_
2	11.31	[M-H]^−^	789.2621	789.2626 (3), 626.1017 (57), 611.1782 (7), 536.2746 (11), 477.1051 (21), 315.0518 (100)	− 1.2	22.3	C_38_H_46_O_18_	Isorhamnetin-Hex-dHex-LinA
3	11.38	[M + 2FA-2H]^2−^	663.3055	1073.5539 (60), 911.5018 (100), 893.4925 (33), 749.4489 (99), 603.3929 (10), 471.3488 (6), 455.2463 (30)	− 0.6	90.4	C_59_H_96_O_27_	C_30_H_48_O_4_-Pen-dHex-Hex_3_
4	11.50	[M-H]^−^	215.1291	215.1291 (100), 197.1188 (94), 153.1285 (6)	− 1.2	2.9	C_11_H_20_O_4_	Unidentified
5	11.81	[M-H]^−^	327.2180	327.2180 (100), 291.1970 (7), 229.1448 (19), 211.1340 (17)	− 1.1	8.8	C_18_H_32_O_5_	Fatty acid / isomer
6	12.28	[M + 2FA-2H]^2−^	736.3354	1099.5719 (3), 1057.5595 (8), 895.5058 (16), 733.4536 (48), 587.3959 (8), 528.2769 (86), 455.3539 (5), 447.2506 (100)	− 1.7	37.3	C_65_H_106_O_31_	C_30_H_48_O_3_-Pen-dHex-Hex_4_
7	12.40	[M-H]^−^	1269.6286	1055.5081 (2), 1011.5177 (26), 849.4646 (62), 687.4123 (100), 541.3545 (25), 409.3118 (6), 183.1030 (11)				Unidentified acylated saponin
8	12.72	[M-H]^−^	329.2339	329.2337 (100), 311.2250 (2), 229.1451 (14), 211.1342 (14)	− 1.8	3.8	C_18_H_34_O_5_	Fatty acid / isomer
9	12.80	[M-H]^−^	301.2030	301.2024 (100), 283.1918 (30), 265.1807 (16)	− 3.0	7.9	C_16_H_30_O_5_	Fatty acid / isomer
10	12.93	[M + H]^+^	1419.7144	783.5031 (7), 637.4460 (27), 471.3467 (34), 453.3362 (100)	0.7	52.9	C_69_H_112_O_30_	Triterpenoid saponin
11	13.00	[M-H]^−^	1271.6392	1271.6389 (100), 1109.5872 (71), 1105.5405 (42), 943.4885 (51), 785.4824 (34), 619.3840 (52), 487.3418 (3), 183.1024 (9)	3.0	71.5	C_63_H_100_O_26_	C_30_H_48_O_5_-Pen-LinA-Hex_3_
12	13.11	[M-H]^−^	287.2232	287.2231 (100)	− 1.4	2.6	C_16_H_32_O_4_	Fatty acid / isomer
13	13.35	[M-H]^−^	431.2280	431.2285 (40), 261.1337 (70), 187.0975 (100)	1.5	23.6	C_21_H_36_O_9_	glycerol-2 × C_9_H_16_O_4_
14	13.67	[M + H]^+^	373.1796	373.1794 (100), 355.1674 (4)	3.4	1.1	C_25_H_26_O_3_	Unidentified aromatic
15	13.76	[M-H]^−^	503.3388	503.3388 (100), 485.3281 (18), 549.3490 (2)	− 1.9	13.9	C_30_H_48_O_6_	Triterpenoid
16	13.87	*[M* + *2NH*_*4*_*]*^*2*+^	*748.3933*	*714.4932 (23), 647.8257 (58), 603.2127 (15), 513.3577 (100), 495.3464 (21), 471.1713 (43), 309.1183 (78), 167.1068 (23)*	− *0.1*	*13.8*	C_71_H_112_O_31_	C_30_H_48_O_5_-AcA-Pen-dHex-LinA-Hex_3_
17	14.07	*[M* + *2NH*_*4*_*]*^*2*+^	*675.3648*	*714.4932 (4), 645.3997 (27), 574.7972 (35), 513.3570 (44), 325.1128 (100), 295.1020 (36), 167.1065 (4), 163.0601 (10)*	− *0.8*	*7.1*	C_65_H_102_O_27_	C_30_H_48_O_5_-AcA-Pen-LinA-Hex_3_
18	14.25	*[M* + *2NH*_*4*_*]*^*2*+^	*675.3644*	*714.4923 (9), 645.3982 (23), 574.7941 (34), 513.3570 (43), 435.3250 (15), 325.1129 (100), 295.1020 (34), 167.1064 (10)*	− *0.2*	*8.2*	C_65_H_102_O_27_	C_30_H_48_O_5_-AcA-Pen-LinA-Hex_3_
19	14.81	*[M* + *2NH*_*4*_*]*^*2*+^	*719.3907*	*1236.6371 (21), 619.3252 (38), 603.2138 (27), 471.1716 (53), 455.3525 (41), 437.3416 (84), 325.1135 (91), 309.1190 (90), 279.1077 (57), 167.1069 (100)*	− *0.3*	*3.0*	C_69_H_110_O_29_	C_30_H_48_O_4_-Pen-dHex-LinA-Hex_3_
20	14.95	*[M* + *2NH*_*4*_*]*^*2*+^	*667.3667*	*714.4921 (20), 645.3984 (16), 566.7996 (69), 513.3572 (100), 495.3463 (24), 325.1132 (23), 309.1184 (86), 279.1076 (71), 167.1064 (21)*	*0.1*	*12.2*	C_65_H_102_O_26_	C_30_H_48_O_5_-AcA-Pen-dHex-LinA-Hex_2_
21	15.33	[M-H]^−^	325.2020	307.1918 (46), 279.1967 (12), 263.2018 (12)	0.3	11.9	C_18_H_30_O_5_	Fatty acid / isomer
22	15.38	[M-H]^−^	1255.6483	1255.6483 (86), 1093.5947 (100), 1075.8553 (30), 927.4960 (35), 769.4899 (56), 603.3910 (60), 471.3486 (4)	− 0.2	10.9	C_63_H_100_O_25_	C_30_H_48_O_4_-Pen-LinA-Hex_3_
23	16.10	[M-H]^−^	287.2230	287.2227 (100), 269.2121 (6)	− 0.7	8.1	C_16_H_32_O_4_	Fatty acid / Isomer
24	16.17	[M + FA-H]^−^	533.3483	487.3430 (100)	0.1	9.9	C_30_H_48_O_5_	Triterpenoid
25	16.26	[M-H]^−^	311.2225	311.2227 (100), 293.2121 (11), 275.2015 (7), 235.1704 (5), 223.1701 (40)	0.8	3.1	C_18_H_32_O_4_	Fatty acid / isomer
26	16.33	[M-H]^−^	649.3741	649.3749 (100), 145.0286 (1)	0.7	16.9	C_39_H_54_O_8_	C_30_H_48_O_5_-CouA
27	16.77	[M-H]^−^	487.3432	487.3431 (100), 469.3327 (14), 443.3529 (2)	− 0.7	19.9	C_30_H_48_O_5_	Triterpenoid
28	17.25	[M-H]^−^	601.3236	601.3242 (28), 431.2291 (100), 261.1345 (41), 243.1243 (8), 225.1125 (2), 187.0979 (52)	− 1.0	10.0	C_30_H_50_O_12_	Glycerol-3 × C_9_H_16_O_4_
29	17.25	[M-H]^−^	487.3430	487.3434 (100)	− 0.3	16.3	C_30_H_48_O_5_	Triterpenoid
30	17.62	[M-H]^−^	593.2740	315.0488 (33), 277.2171 (100), 259.0226 (4), 241.0117 (27), 223.0013 (7), 152.9951 (11)	− 1.2	5.2	C_27_H_47_O_12_P	Octadecatrienoyl-glycero-3-PI
31	17.62	[M-H]^−^	474.2630	474.2630 (100), 400.2257 (14), 382.2157 (1), 214.0500 (0.5), 171.0065 (8), 152.9952 (2)	− 0.5	4.5	C_23_H_42_NO_7_P	N-octadecatrienoyl-glycero-3-PE
32	18.07	[M-H]^−^	593.2731	413.2097 (7), 315.0486 (31), 277.2168 (100), 241.0116 (38), 152.9951 (6)	0.3	9.2	C_27_H_47_O_12_P	Octadecatrienoyl-glycero-3-PI
33	18.37	[M-H]^−^	518.2533	431.2204 (100), 277.2176 (14), 171.0054 (6), 152.9949 (75)	− 1.7	4.1	C_23_H_42_NO_9_P	Octadecatrienoyl-glycero-3-PS
34	18.37	[M-H]^−^	259.2120	269.2118 (100), 251.2015 (11)	0.8	23.1	C_16_H_30_O_3_	Fatty acid / isomer
35	18.54	[M-H]^−^	474.2428	474.2629 (4), 277.2173 (100), 214.0484 (2), 196.0381 (6)	− 0.4	28.4	C_23_H_42_NO_7_P	Octadecatrienoyl-glycero-3-PE
36	18.99	[M-H]^−^	595.2895	595.2894 (30), 315.0491 (32), 279.2330 (100), 241.0121 (26), 223.0016 (8), 152.9955 (10)	− 1.0	20.2	C_27_H_49_O_12_P	Octadecadienoyl-glycero-3-PI
37	19.11	[M-H]^−^	476.2788	476.2785 (100), 402.2419 (13), 171.0065 (7), 152.9955 (3)	− 1.1	4.7	C_23_H_44_NO_7_P	N-octadecadienoyl-glycero-3-PE
38	19.46	[M-H]^−^	595.2893	595.2894 (55), 415.2260 (8), 315.0492 (31), 279.2330 (100), 241.0122 (36), 152.9955 (6)	− 0.7	5.8	C_27_H_49_O_12_P	Octadecadienoyl-glycero-3-PI
39	20.03	[M-H]^−^	571.2881	571.2877 (46), 315.0479 (46), 255.2318 (100), 241.0110 (35), 223..0005 (9), 152.9945 (13)	1.5	7.4	C_25_H_49_O_12_P	Hexadecanoyl-glycero-3-PI
40	20.11	[M-H]^−^	431.2197	431.2193 (17), 277.2163 (23), 171.9989 (5), 152.9949 (100)	1.7	14.4	C_21_H_37_O_7_P	Octadecatrienoyl-glycero-3-P
41	20.35	[M-H]^−^	445.2369	277.2176 (100), 185.0226 (1), 167.0115 (0.3)	− 1.9	12.6	C_22_H_39_O_7_P	Methylated octadecatrienoyl-glycero-3-P
42	20.47	[M-H]^−^	571.2895	391.2265 (11), 315.0496 (41), 255.2333 (100), 241.025 (44), 152.9958 (8)	− 1.0	12.9	C_25_H_49_O_12_P	Hexadecanoyl-glycero-3-PI
43	20.55	[M-H]^−^	445.2366	277.2175 (100), 167.0119 (3)	− 1.3	7.9	C_22_H_39_O_7_P	Methylated octadecatrienoyl-glycero-3-P
44	20.83	[M-H]^−^	507.2738	279.2333 (100), 277.0330 (5), 152.9957 (2)	− 1.9	5.3	C_24_H_45_O_9_P	Octadecadienoyl-glycero-3-PG
45	21.41	[M-H]^−^	597.3055	597.3052 (65), 417.2421 (9), 315.0494 (33), 281.2489 (100), 241.0125 (38), 152.9958	− 1.6	10.0	C_27_H_51_O_12_P	Octadecenoyl-glycero-3-PI
46	21.82	[M-H]^−^	433.2351	433.2346 (27), 279.2316 (28)171.0055 (7), 152.9944 (100)	2.2	4.7	C_21_H_39_O_7_P	Octadecadienoyl-glycero-3-P
47	22.18	[M-H]^−^	447.2527	279.2136 (100), 185.0229 (1)	− 2.2	8.1	C_22_H_41_O_7_P	Methylated octadecadienoyl-glycero-3-P
48	22.51	[M-H]^−^	447.2527	279.2136 (100), 185.0232 (1), 167.0122 (3)	− 2.2	5.0	C_22_H_41_O_7_P	Methylated octadecadienoyl-glycero-3-P
49	24.17	[M-H]^−^	423.2520	423.2526 (4), 255.2331 (100), 167.0118 (3)	− 0.8	10.8	C_20_H_41_O_7_P	Methylated hexadecanoyl-glycero-3-P
50	25.12	[M-H]^−^	449.2668	449.2661 (3), 281.2474 (100), 185.0216 (1), 167.0109 (3)	1.4	6.4	C_22_H_43_O_7_P	Methylated octadecenoyl-glycero-3-P

*FA* formic acid; *Hex* hexose; *dHex* deoxyhexose; *Pen* pentose; *AcA* acetic acid; *CouA* coumaric acid; *LinA* (E)-linalool-1-oic acid; *PE* phosphoethanolamine; *PG* phosphoglycerol; *PI* phosphoinositol; *PS* phosphoserine; *P* phosphate; *italic* – data from the LC–MS analysis, in which the mobile phase contained ammonium formate buffer, as described in the Materials and methods.

### Biomarkers of oxidative stress in plasma

**Fig. 4:**
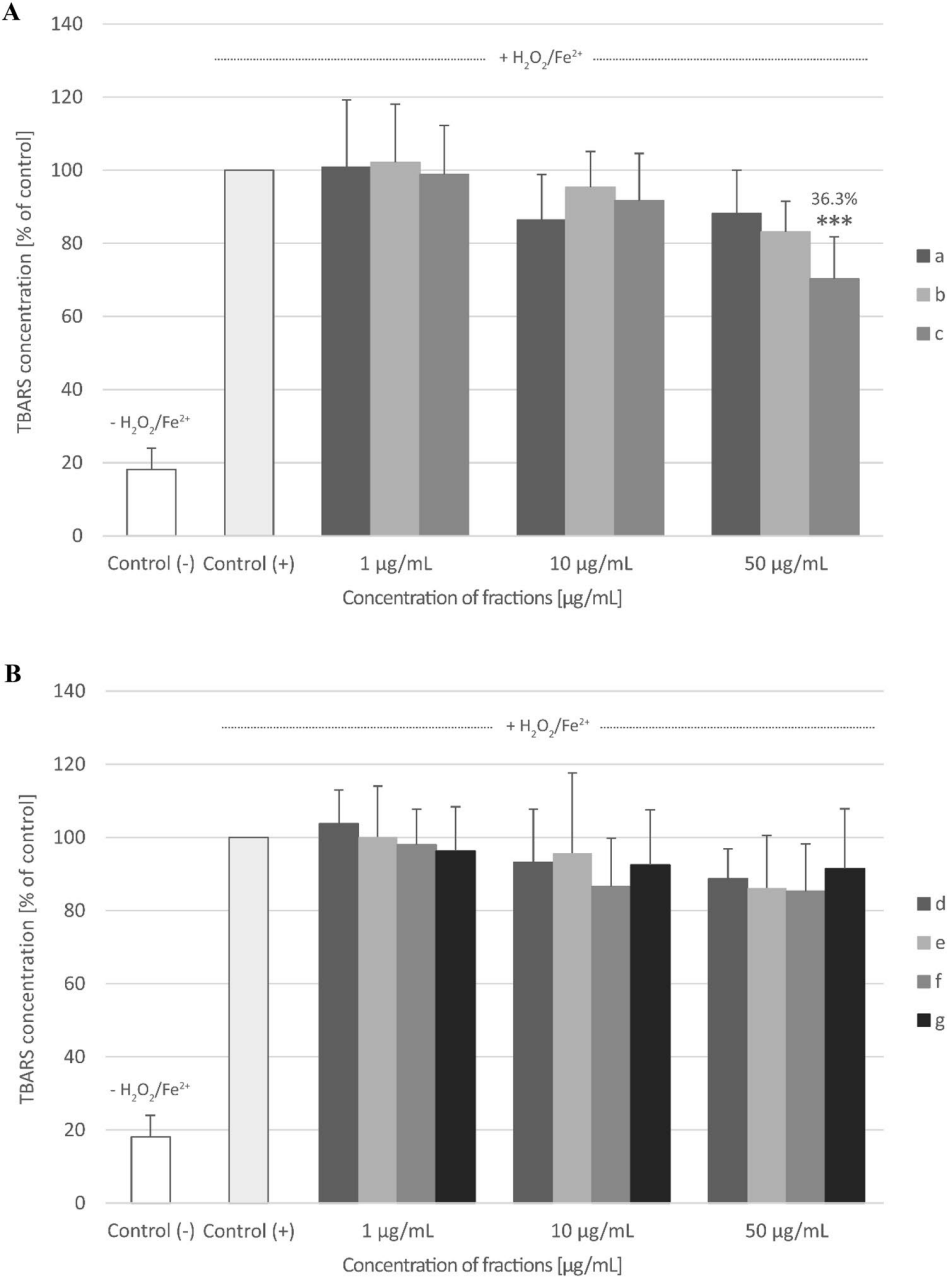
The effect of three fractions (a, b, and c) from raw (**A**) and four fractions (d, e, f, and g) from roasted (**B**) sea buckthorn seeds on the level of thiobarbituric acid reactive substances (TBARS) in human plasma treated with an oxidative stress inducer (Fe^2+^/H_2_O_2_) (n = 9). Control (+) and test samples (0.5–50 μg/mL) were incubated (30 min, 37 °C) with Fe^2+^/H_2_O_2_. The results are presented as % of control (+). The data are expressed as means ± SD. One way-ANOVA with Tukey’s test was used to assess the differences between the control and the test samples. The results were considered significant at *p* < 0.05 (***p < 0.001). The numbers above significant results are % of lipid peroxidation inhibition. The difference between control (−) and control (+) was statistically significant (*p* < 0.001).

**Fig. 5:**
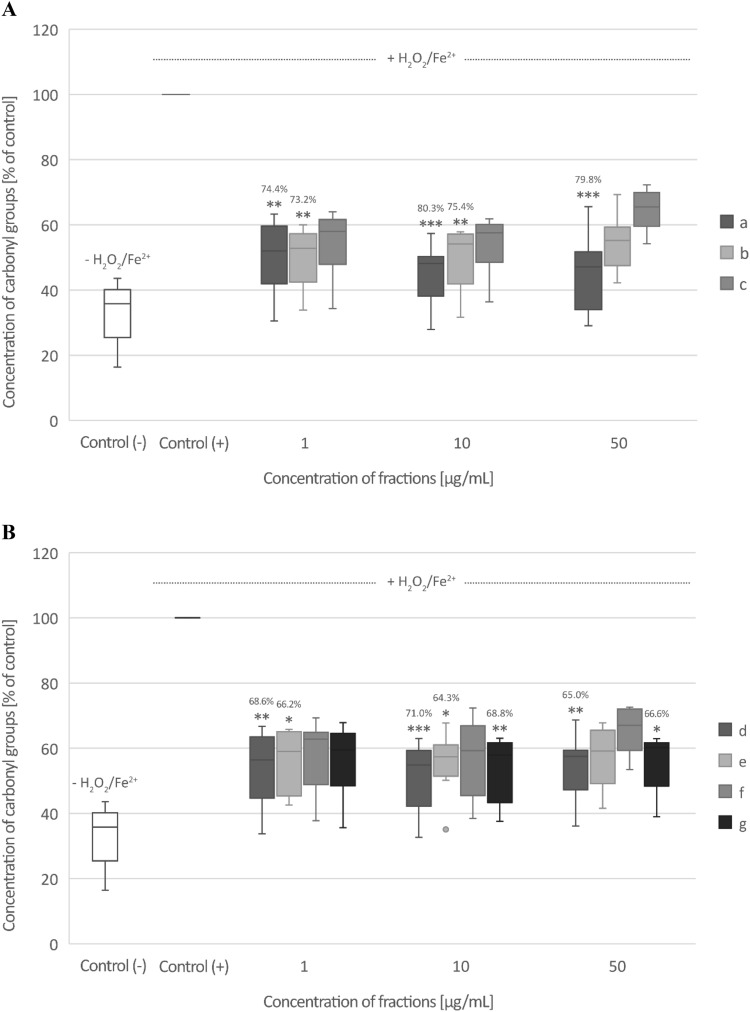
The effect of three fractions (a, b, and c) from raw (**A**) and four fractions (d, e, f, and g) from roasted (**B**) sea buckthorn seeds on the level of carbonyl (CO) groups in human plasma treated with an oxidative stress inducer (Fe^2+^/H_2_O_2_) (n = 8). Control (+) and test samples (0.5–50 μg/mL) were incubated (30 min, 37 °C) with Fe^2+^/H_2_O_2_. The results are presented as % of control (+). The data are expressed as medians and interquartile ranges. Kruskal–Wallis test was used to assess the differences between the control and the test samples. The results were considered significant at *p* < 0.05 (*p < 0.05; **p < 0.01; *** *p* < 0.001). The numbers above significant results are % of protein carbonylation inhibition. The difference between control (−) and control (+) was statistically significant (*p* < 0.001).

**Fig. 6:**
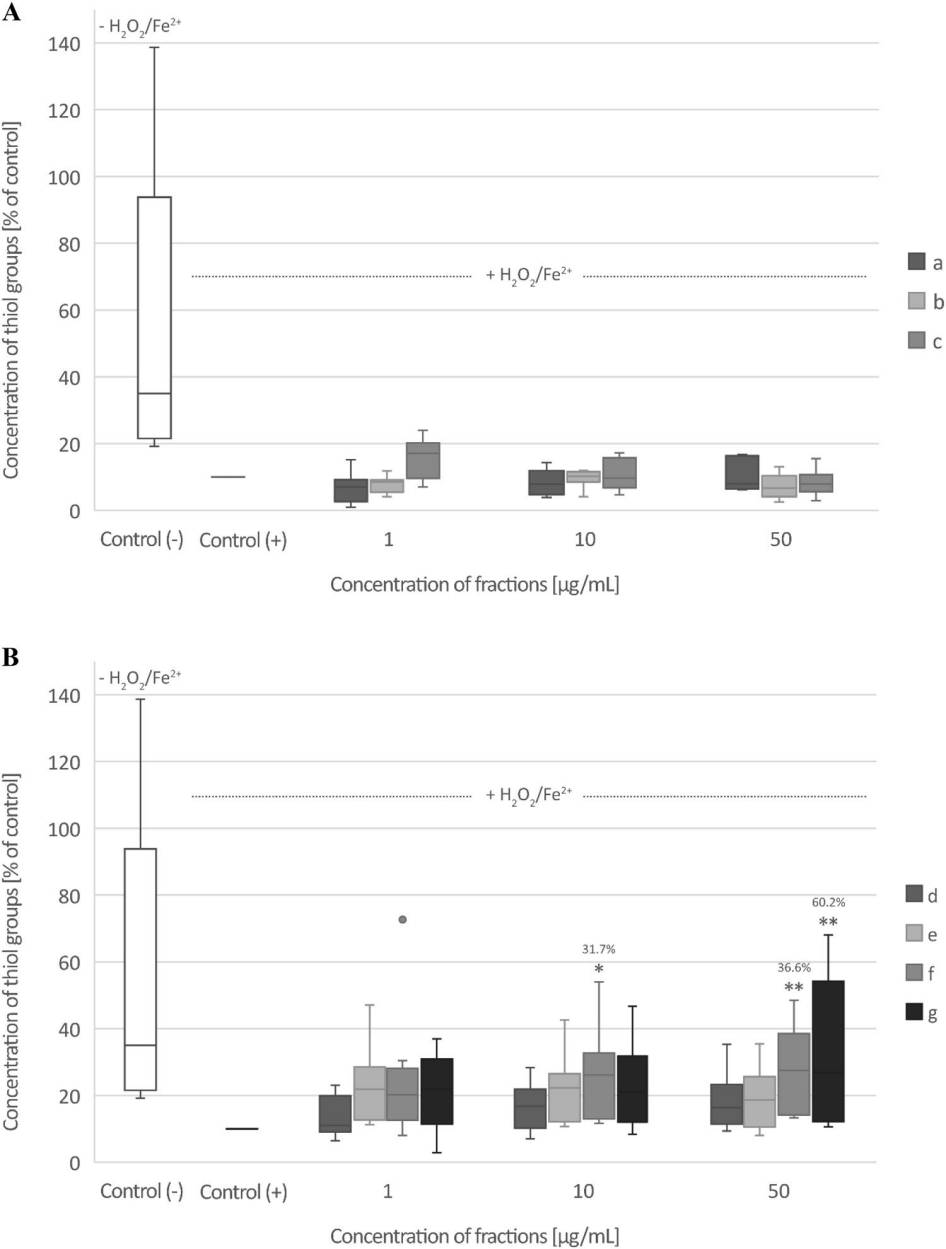
The effect of three fractions (a, b, and c) from raw (**A**) and four fractions (d, e, f, and g) from roasted (**B**) sea buckthorn seeds on the level of thiol groups in human plasma treated with an oxidative stress inducer (Fe^2+^/H_2_O_2_) (n = 9). Control (+) and test samples (0.5–50 μg/mL) were incubated (30 min, 37 °C) with Fe^2+^/H_2_O_2_. The results are presented as % of control (+). The data are expressed as medians and interquartile ranges. Kruskal–Wallis test was used to assess the differences between the control and the test samples. The results were considered significant at *p* < 0.05 (*p < 0.05, **p < 0.01). The numbers above significant results are % of thiol groups oxidation inhibition. The difference between control (−) and control (+) was statistically significant (*p* < 0.001).

Only fraction c (from raw seeds) at the highest used concentration—50 µg/mL significantly inhibited plasma lipid peroxidation induced by H_2_O_2_/Fe^2+^ (% of inhibition—36.3%) (Fig. 4A,B). All three fractions (a, b, and c) from raw seeds and all four fractions (d, e, f, and g) from roasted seeds at all tested concentrations (1–50 µg/mL) decreased plasma protein carbonylation stimulated by H_2_O_2_/Fe^2+^, but this action was not always statistically significant (Fig. 5A,B). None of the tested fractions from raw seeds changed the level of thiol groups in plasma treated with H_2_O_2_/Fe^2+^ (Fig. 6A). However, the roasted seed fractions: fraction f (10 and 50 µg/mL) and g (50 µg/mL) significantly inhibited thiol group oxidation stimulated by H_2_O_2_/Fe^2+^ (Fig. 6B).

**Fig. 7:**
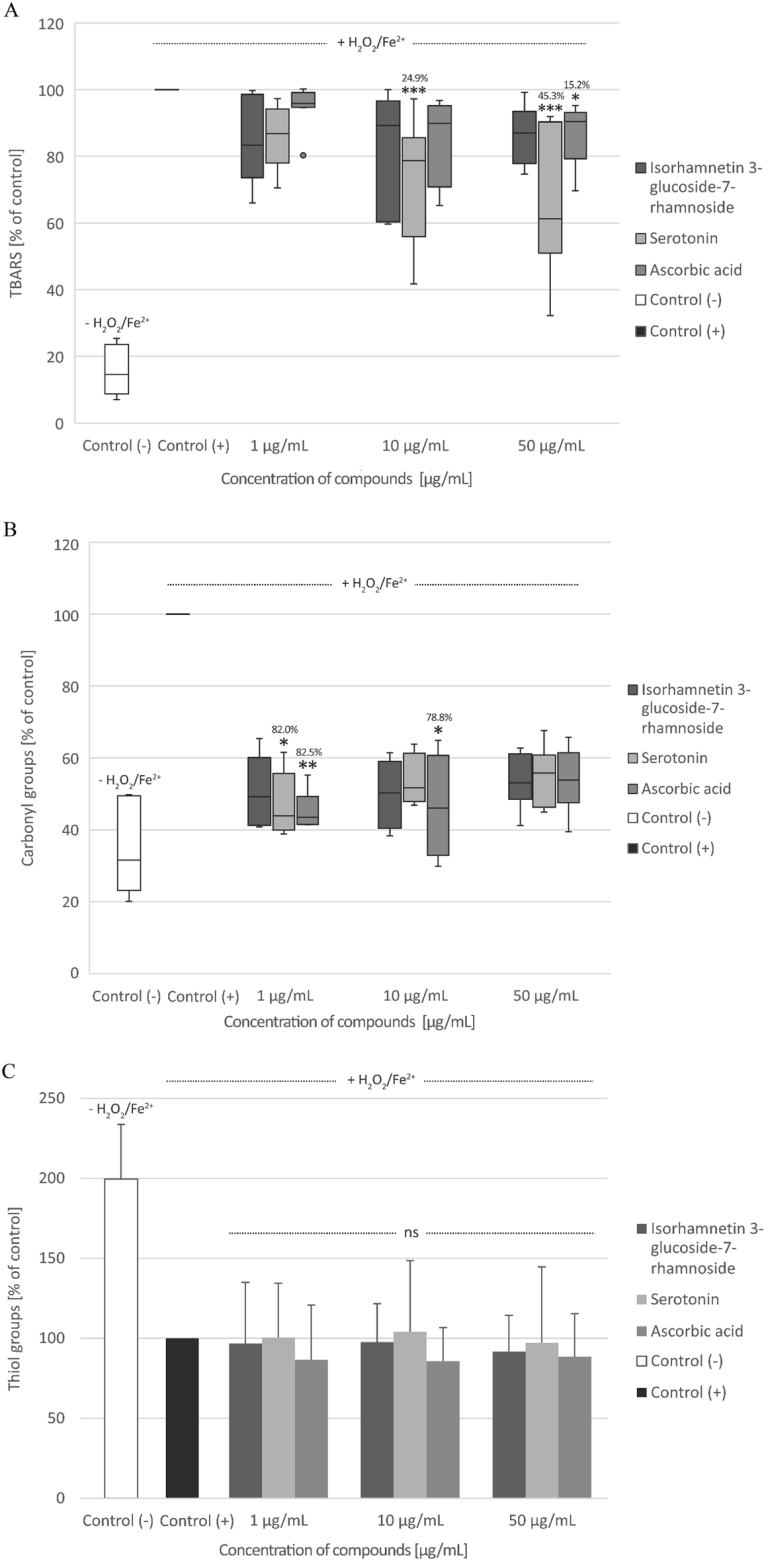
Effect of isorhamnetin 3-*O*-β-glucoside7-*O*-α-rhamnoside (1–50 µg/mL) and serotonin (1–50 µg/mL) on the level of thiobarbituric acid reactive substances (TBARS) (**A**), carbonyl (CO) groups (**B**), and thiol groups (**C**) in human plasma treated with an oxidative stress inducer (Fe^2+^/H_2_O_2_) (n = 9 (A); n = 10 (B); n = 9 (C)). Control (+) and test samples (1–50 μg/mL) were incubated (30 min, 37 °C) with Fe^2+^/H_2_O_2_. The results are presented as % of control (+). The data are expressed as medians and interquartile ranges (A, B) or means ± SD (C). Kruskal–Wallis test (A and B) or one-way ANOVA (C) was used to assess the differences between the control and the test samples. The results were considered significant at *p* < 0.05 (*p < 0.05, **p < 0.01, ***p < 0.001). The numbers above significant results are % of inhibition. The differences between control (−) and control (+) were statistically significant (*p* < 0.001).

Plasma lipid peroxidation stimulated by H_2_O_2_/Fe^2+^ was reduced in the presence of isorhamnetin 3-*O*-β-glucoside7-*O*-α-rhamnoside and serotonin (at concentrations: 1, 10, and 50 µg/mL), but this process was not always statistically significant (Fig. 7A). Only two concentrations of serotonin (10 and 50 µg/mL) and the highest concentration (50 μg/mL) of ascorbic acid significantly inhibited lipid peroxidation by about 24.9, 45.3, and 15.2%, respectively (Fig. 7A). Both, isorhamnetin 3-*O*-*β*-glucoside7-*O*-α-rhamnoside and serotonin reduced protein carbonylation induced by H_2_O_2_/Fe^2+^, but this action was not always statistically significant (Fig. 7B). Moreover, none of the tested compounds had any effect on the oxidation of thiol groups in plasma treated with H_2_O_2_/Fe^2+^ (Fig. 7C).

### Coagulation times (PT, TT, and APTT)

None of the fractions from raw and roasted seeds significantly impacted the coagulation times measured in plasma at both used concentrations (10 and 50 µg/mL) (data not presented).

### A comparison of the antioxidative activity of various fractions

Effects of various tested fractions (at the highest used concentration—50 µg/mL) on oxidative stress in human plasma treated with H_2_O_2_/Fe^2+^ were compared in Table 4. Out of all the tested fractions, fraction g (isolated from roasted seeds) demonstrated the strongest antioxidant properties. It reduced plasma protein carbonylation and oxidation of protein thiol groups. Moreover, two fractions (a and c) from raw seeds and two fractions (d and f) from roasted seeds had stronger antioxidant activity than other fractions (b and e). In addition, thermal processing increased the antioxidant activity of raw seeds—only fractions f and g from raw seeds inhibited thiol groups oxidation induced by H_2_O_2_/Fe^2+^. On the other hand, only fraction c from raw seeds significantly decreased lipid peroxidation.

**Table 4:** Comparative effects of three fractions (a, b, and c; 50 µg/mL) from raw sea buckthorn seeds (no thermal processing) and four fractions (d, e, f, and g; 50 µg/mL) from roasted sea buckthorn seeds (thermally processed) on oxidative stress in plasma treated with H_2_O_2_/Fe^2+^.

	Raw seeds			Roasted seeds			
	Fraction a	Fraction b	Fraction c	Fraction d	Fraction e	Fraction f	Fraction g
Lipid peroxidation	No effect	No effect	**Antioxidant activity**	No effect	No effect	No effect	No effect
Protein carbonylation	**Antioxidant activity**	No effect	No effect	**Antioxidant activity**	No effect	No effect	**Antioxidant activity**
Thiol oxidation	No effect	No effect	No effect	No effect	No effect	**Antioxidant activity**	**Antioxidant activity**

## Discussion

While there are many publications describing the composition of sea buckthorn seed oil, its carotenoids, tocochromanols, and sterols, the knowledge about other groups of natural products present in the seeds is lacking. Seed flavonoids have been characterized quite well, but the data concerning other groups of metabolites are scarce. Our LC–MS analyses showed that serotonin was the dominant constituent of fractions c and f (Fig. 1, Table 1), it was tentatively identified on the basis of its HRMS and UV spectra. The presence of serotonin in the bark and other parts of sea buckthorn, including seeds, is quite well documented in the literature^15–19^. A putative sulfur-containing dipeptide and a putative indolylacetic acid hexoside seemed to be other major constituents of fraction f, and were also present in fraction c. It cannot be excluded that the dipeptide is *γ*-glutamyl-S-methylcysteine. The detected compound has the MS/MS spectrum similar to those reported by Joshi et al.^20^, and Chen et al.^21^. This would be probably the first report about its presence in sea buckthorn. γ-glutamyl-S-methylcysteine was previously found in the seeds of certain legumes, which may increase the reliability of identification^20,22–25^. It also seems to be probable that the detected putative derivative of indolylacetic acid is 7-hydroxy-2-oxindole-3-acetic acid 7'-*O*-3-β-glucoside, a product of auxin metabolism, which was isolated from maize seedlings; its UV spectrum is quite similar to that of the compound from fractions f and c (which has maxima at 255 and 296 nm)^26^. Fractions c and f, as well as b and e, also contained different B-type proanthocyanidins. The detected compounds were similar to those found in seeds of *H. rhamnoides* ssp. *sinensis* by Fan et al.^27^.

Fractions b and e consisted mainly of numerous glycosides of kaempferol, quercetin, and isorhamnetin. 3-*O*-dihexoside-7-*O*-deohyhexosides of these aglycones (most probably 3-*O*-sophoroside-7-*O*-rhamnosides), as well as isorhamnetin 3-*O*-β-glucoside-7-*O*-α-rhamnoside were dominant constituents of both preparations (Fig. 2, Table 2). The presence of such flavonoid glycosides in sea buckthorn seeds is confirmed by publications of other research groups^12,28,29^. The fractions also contained many flavonol tri- and diglycosides acylated with (2*E*)-2,6-dimethyl-6-hydroxy-2,7-octadienolic acid ((*E*)-linalool-1-oic acid), phenolic acids (sinapic, ferulic), or both of them (Fig. 2, Table 2). It seems that such compounds are characteristic for sea buckthorn seeds, as many similar kaempferol glycosides were previously isolated from seeds of *H. rhamnoides* ssp. *sinensis*^28,30,31^. Apart from flavonol glycosides, the fractions also contained catechin, epicatechin, phenolic acid derivatives, sesquiterpenoid glycosides, vomifoliol hexoside-pentoside, alkaloids, triterpenoid saponins, as well as some unidentified constituents. Most of these compounds occurred in small amounts. The detected vomifoliol glycoside is probably vomifoliol 3’-*O*-*β*-D-apiofuranosyl-(1 → 6)-*β*-D-glucopyranoside, purified from seeds of *H. rhamnoides* ssp. *sinensis* by Gao et al.^15^. Regarding alkaloids, four compounds were detected. Two of them were preliminarily identified as β-carboline alkaloids, harmalol and harmol. Our data partly corroborate the results of Michel^33^, who used LC-HRMS to detect two putative β-carboline alkaloids in sea buckthorn fruit; one of them was tentatively identified as harmalol. Another alkaloid (Table 2, compound 18), a coumaric acid derivative found in fractions b and e, is most likely 4-[*p*-coumaroylamino]butan-1-ol, a compound earlier purified from the seeds of *H. rhamnoides* ssp. *sinensis*^34^.

Fractions a and d contained mainly triterpenoid saponins. Most of these compounds, including major constituents of the fractions, were acylated with (2*E*)-2,6-dimethyl-6-hydroxy-2,7-octadienolic acid, some of them were also acetylated (Fig. 3, Table 3). Small amounts of unacylated saponins were also present in fractions b and e. The same or similar compounds were found earlier in sea buckthorn leaves^35,36^. Six triterpenoid saponins were also previously isolated from *H. rhamnoides* ssp. *sinensis* seeds^32^, but they were different from those described in the current work. The fractions also contained free triterpenoids (Table 3), but in a smaller number than other parts of sea buckthorn^35,37^. Small amounts of diverse lysophospholipids were also detected, such as lysophosphatidylethanolamines, lysophosphatidylinositols, lysophosphatidylserines, lysophophatidylglycerols, lysophosphatidic acids. Moreover, both fractions contained glycerophospho N-acylethanolamines (GP-NAE, NA-GPE), similar to compounds reported from seeds of hemp or hazel^38,39^.

Oxidative stress is known as one of the major contributors in various diseases, including cardiovascular diseases and cancer. In general, it is not recommended to evaluate the antioxidant activity of plant preparations by only a single method due to the complex nature of phytocompounds. Therefore, in the present in vitro study, three assays (including thiol and carbonyl groups’ determination, and lipid peroxidation) were used to study oxidative stress in human plasma.

Thermal processing has various effects on the antioxidant properties of fruits, vegetables, and seeds. Heat treatment might decrease antioxidant activity, but in some cases, the effect can be opposite—antioxidant activity can improve. The outcome is largely dependent on the method of processing, temperature/power, and time^40–44^. In addition, the stability of phytocompounds during thermal processing depends on the type of compound and plant material in which the compound is encased during heating^45–48^.

We reported for the first time that three fractions from raw sea buckthorn seeds and four fractions from roasted sea buckthorn seeds differed in terms of antioxidant properties, in an experimental model based on human plasma. The easiest way to explain these differences relates to the different chemical profiles of each of the used fractions. In addition, we suggest that thermal processing can increase the antioxidant activity of raw sea buckthorn seeds.

Fractions a (from raw seeds) and d (from roasted seeds) were very similar in composition and consisted mainly of various triterpenoid saponins. Both showed comparable antioxidant activity by decreasing protein carbonylation. Other researchers also reported that triterpenoid saponins possess antioxidant potential, which is in line with our results^49–51^. For instance, Liu et al.^50^ noted that triterpenoid saponins from the roots of *Glycyrrhiza uralensis* decreased Fe^2+^/cysteine-induced liver microsomal lipid peroxidation, while Yang et al.^52^ reported the antioxidant potential of triterpenoids isolated from sea buckthorn branch bark. Our earlier results also demonstrated that a triterpenoid fraction from sea buckthorn twigs reduces lipid peroxidation and protein carbonylation in plasma treated with H_2_O_2_/Fe^2+^^53^. The small differences in the activity of fractions a and d might be caused by small changes in composition induced by thermal processing.

Only fractions f and g from roasted seeds significantly inhibited thiol groups oxidation. These observations are similar to other studies, where thermal processing improved the antioxidant activity of seeds. For example, roasting increased the antioxidant capacity of coffee beans^54^ and peanuts (*Arachis hypogaea* L.)^55^.

Serotonin, a dominant constituent of fractions c and f showed the strongest antioxidant potential out of the three tested compounds by inhibiting both lipid peroxidation and protein carbonylation. Serotonin is an ancient indoleamine with important functions in both plants and animals. In the past, it was known mainly for its role as a neurotransmitter, but recently, other functions of serotonin, including its antioxidant activity, are being explored in depth^56^. Azouzi et al.^57^ proposed that serotonin prevents lipid peroxidation by binding to lipid bilayers, preferentially to lipids with unsaturation on both alkyl chains. This would allow it to trap hydroxyl radicals before they have the chance to interact with lipids. Moreover, serotonin’s hydroxyl group might contribute to its reactive oxygen species (ROS)-scavenging capacity.

Various glycosides of isorhamnetin, kaempferol, and quercetin (especially isorhamnetin 3-*O*-glucoside-7-*O*-rhamnoside) were the main constituents of fractions b and e. Isorhamnetin 3-*O*-glucoside-7-*O*-rhamnoside showed some degree of antioxidant activity, but comparatively stronger activity of fractions b and f suggest that the combined action of other flavonol glycosides also contribute to their antioxidant properties. Skalski et al.^14^ also noted that these compounds isolated from sea buckthorn berries have antioxidant potential in vitro.

Different tested fractions, including b, c, e, and f contained B-type proanthocyanidins as well. B-type proanthocyanidins have a proven antioxidant potential which might enhance the antioxidant properties of the tested fractions^58,59^.

In addition, our present results may suggest that tested fractions are often more effective antioxidant factors than pure chemical compounds. These results may also indicate that various compounds have synergistic actions.

Oxidative stress can influence hemostasis, including the coagulation process. Moreover, it is known that thermal processing can change not only the antioxidant activity of seeds but also other biological properties; for example, the anti-inflammatory potential of coffee beans was decreased by high-intensity roasting^60^. However, in our previous study on the extracts from raw and roasted sea buckthorn seeds, both used extracts (0.5–50 µg/mL) had no effect on plasma coagulation times^8^. In the present study, the seven used fractions (a-g) from the seeds also did not change the coagulation activity of plasma in vitro.

Despite the promising in vitro antioxidant activity of fractions from sea buckthorn seeds, poor bioavailability of most phenolic compounds might be an obstacle in achieving the same results in vivo*.* Suomela et al.^61^ reported that administration of 0.4 g of sea buckthorn flavonol extract (containing 78 mg of flavonol aglycones) daily for 4 weeks failed to reduce CVDs risk markers and oxidative stress in healthy subjects, which suggests that a higher dosage might be needed to induce significant effects^61^. The bioavailability of most triterpenoid saponins is relatively low, however, it can sometimes be improved by microbial biotransformation^62^. Thermal processing can be a way of improving the bioaccessibility and bioavailability of various compounds by detaching them from protein complexes or releasing them from the food matrix^63,64^. For example, phenolic compounds from highland barley had higher bioaccessibility after thermal processing^64^.

The phenolic content of sea buckthorn berries was changed after enzymatic digestion; their antioxidant properties were significantly enhanced by this process^65^. Results of Baeza et al.^66^ also found that various metabolites of phenolic compounds have stronger biological activity than their precursors.

Our previous studies^8^ together with the present experiments indicate that sea buckthorn seeds are a rich source of antioxidants; the observed properties can be linked not only with the presence of phenolic compounds but also with triterpenoids. In addition, we suggest that not only raw but also roasted sea buckthorn seeds can be a new source of natural antioxidants (as functional food or supplements), beneficial in the prevention and treatment of various diseases associated with oxidative stress. However, more research is needed to provide a better understanding of the antioxidative mechanism of bioactive compounds of sea buckthorn seeds after thermal processing and determine their effectiveness in vivo.

## Data Availability

The datasets generated and/or analyzed during the current study are available from the corresponding author on reasonable request.

## Abbreviations

5-HT: 5-Hydroxytryptamine
APTT: Activated partial thromboplastin time
CVDs: Cardiovascular diseases
PT: Prothrombin time
ROS: Radical oxygen species
TBARS: Thiobarbituric acid reactive substances
TT: Thrombin time
